# A Prime-Boost Vaccination Strategy in Cattle to Prevent Foot-and-Mouth Disease Using a “Single-Cycle” Alphavirus Vector and Empty Capsid Particles

**DOI:** 10.1371/journal.pone.0157435

**Published:** 2016-06-13

**Authors:** Maria Gullberg, Louise Lohse, Anette Bøtner, Gerald M. McInerney, Alison Burman, Terry Jackson, Charlotta Polacek, Graham J. Belsham

**Affiliations:** 1 DTU National Veterinary Institute, Technical University of Denmark, Lindholm, Kalvehave, Denmark; 2 Department of Microbiology, Tumor and Cell Biology, Karolinska Institutet, Stockholm, Sweden; 3 The Pirbright Institute, Pirbright, Woking, Surrey, United Kingdom; Institut National de la Santé et de la Recherche Médicale (INSERM), FRANCE

## Abstract

Foot-and-mouth disease (FMD) remains one of the most economically important infectious diseases of production animals globally. Vaccination can successfully control this disease, however, current vaccines are imperfect. They are made using chemically inactivated FMD virus (FMDV) that is produced in large-scale mammalian cell culture under high containment conditions. Here, we have expressed the FMDV capsid protein precursor (P1-2A) of strain O1 Manisa alone or with the FMDV 3C protease (3C^pro^) using a “single cycle” packaged alphavirus self-replicating RNA based on Semliki Forest virus (SFV). When the FMDV P1-2A was expressed with 3C^pro^ then processing of the FMDV capsid precursor protein is observed within cells and the proteins assemble into empty capsid particles. The products interact with anti-FMDV antibodies in an ELISA and bind to the integrin α_v_β_6_ (a cellular receptor for FMDV). In cattle vaccinated with these rSFV-FMDV vectors alone, anti-FMDV antibodies were elicited but the immune response was insufficient to give protection against FMDV challenge. However, the prior vaccination with these vectors resulted in a much stronger immune response against FMDV post-challenge and the viremia observed was decreased in level and duration. In subsequent experiments, cattle were sequentially vaccinated with a rSFV-FMDV followed by recombinant FMDV empty capsid particles, or *vice versa*, prior to challenge. Animals given a primary vaccination with the rSFV-FMDV vector and then boosted with FMDV empty capsids showed a strong anti-FMDV antibody response prior to challenge, they were protected against disease and no FMDV RNA was detected in their sera post-challenge. Initial inoculation with empty capsids followed by the rSFV-FMDV was much less effective at combating the FMDV challenge and a large post-challenge boost to the level of anti-FMDV antibodies was observed. This prime-boost system, using reagents that can be generated outside of high-containment facilities, offers significant advantages to achieve control of FMD by vaccination.

## Introduction

Foot-and-mouth disease (FMD) remains one of the most feared virus infections of farm animals. The virus can infect cattle, pigs, sheep and goats plus about 70 wildlife species. The disease continues to affect over 100 countries and has a huge economic impact globally which has been estimated at about US$10,000,000,000 annually [1]. Indeed outbreaks within individual countries, that have highly developed agriculture, can also have economic consequences of this severity; for example, the epizootic in the UK, in 2001, affected more than 2000 farms and resulted in the slaughter of several million animals [2].

The disease is caused by infection with foot-and-mouth disease virus (FMDV); this is the prototypic member of the *Aphthovirus* genus within the family *Picornaviridae*. Like other picornaviruses, FMDV has a single-stranded, positive sense, RNA genome that is enclosed within a protein shell comprised of 60 copies of 4 different structural proteins, VP1, VP2, VP3 and VP4. Only VP1-VP3 are surface exposed on the virus particles, whereas the VP4 is internal [3]. The capsid serves to protect the RNA genome when the virus is outside of the cell. In addition, the capsid proteins facilitate virus entry (by binding to certain integrin receptors, e.g. α_v_β_6_) [4,5] and delivery of the genome into the cytoplasm of the cell where translation and replication of the viral RNA takes place and new virus particles are formed [6]. The surface exposed capsid proteins are also recognized by the immune system of infected animals and induce neutralizing antibodies, the key requirement for protection against infection by this virus (reviewed in [7]).

There are seven different serotypes of FMDV, namely O, A, C, SAT 1, SAT 2, SAT 3 and Asia-1. Infection by, or vaccination against, one serotype does not confer protection against other serotypes. Current vaccines against FMD are based on chemically inactivated FMDV, grown in cell culture, and used with a suitable adjuvant [7,8]. The FMDV antigen is generally purified to remove the non-structural proteins (NSPs) produced by the virus since the lack of antibodies to the NSPs can enable vaccinated animals to be distinguished from infected animals. Current vaccines confer relatively short-term immunity (vaccination is often performed 2–3 times per year in countries where this is permitted) and require maintenance of a cold-chain. Vaccination against FMDV helped to free Europe from the disease in the 1960’s and 1970’s but currently vaccination against FMDV is not permitted in Europe except in the face of an outbreak. However, the scale of the FMDV outbreak in the UK in 2001 has encouraged the search for improved vaccines against this disease [9]. The most successful viral vaccines have been based on “live” attenuated viruses (e.g. for smallpox, rinderpest and poliovirus) but no such “live” FMDV vaccine, has been found to be satisfactory.

It is possible to produce FMDV empty capsid particles (which comprise 60 copies of VP0 (uncleaved VP4 and VP2), VP3 and VP1) by expressing the capsid protein precursor P1-2A in cells along with the FMDV 3C protease (3C^pro^) [10,11,12,13]. This viral protease processes the P1-2A precursor to VP0, VP3 and VP1 (and the 2A peptide) and these proteins “self-assemble” into particles. The P1-2A precursor is modified on the N-terminal Gly (G) residue by the cellular myristoylation machinery [14]. This post-translational modification is essential for the assembly and/or stability of most picornavirus capsids [10,15]. Recombinant empty capsid particles produced in this way may represent a good alternative to the conventional FMDV vaccine since they are non-infectious and can be made outside of high containment facilities and may also be modified to enhance their stability [11,16,17,18,19]. However, vaccines based on these empty capsid particles may still be expected to suffer from some of the same shortcomings, (e.g. in terms of duration of immunity), as the current inactivated vaccines.

Infectious vaccines (such as live attenuated viruses or vaccines delivered by modified viral vectors) are able to produce virus antigens within infected cells and then these can be presented on the cell surface to the immune system. The expression of serotype A FMDV capsid proteins using an infectious adenovirus vector has been described and good protection against challenge has been achieved in cattle [20]. However, analogous adenoviruses expressing serotype O capsid proteins have been less successful [21]; it is important to note that serotype O FMDV is the most common globally. High doses of the recombinant adenoviruses are required to induce protection against FMDV. It may be that these viral vectors, which express the RNA transcripts from within the nucleus of mammalian cells, are not optimal [9].

Certain cytoplasmic RNA viruses have been modified for use as expression vectors (see [22]). The alphaviruses, like picornaviruses, have a positive sense RNA genome, however, in addition to the genomic RNA, alphaviruses also produce a sub-genomic RNA that encodes the viral structural proteins. This subgenomic RNA is not required for RNA replication and hence can be modified without impeding this process. Expression vectors based on Semliki Forest virus (SFV) have been described [22] and derivatives that use a “split helper” system have been developed [23]. In this system, packaging of the modified SFV genomic RNA transcripts is achieved by co-expression of two separate helper RNAs encoding the capsid and envelope structural proteins. Only the modified genomic RNAs, containing a packaging signal, are incorporated into progeny SFV particles while the mRNAs encoding the capsid and spike proteins are translated to produce the structural proteins. The virus particles made in this way can infect cells but are unable to generate new infectious progeny; thus only a single round of cell infection occurs.

In this study, RNA sequences encoding a serotype O FMDV capsid protein precursor (P1-2A), with the FMDV 3C^pro^, have been expressed from recombinant SFV (rSFV) vectors in cells and FMDV empty capsid particles were produced. Vaccination of cattle with these rSFV-FMDV vectors primed a strong anti-FMDV immune response that was observed following a booster vaccination, with FMDV empty capsid particles. This resulted in a complete block on FMDV circulation within the animals following virus challenge and the animals were protected against disease.

## Materials and Methods

### Plasmid construction

**Fig 1:**
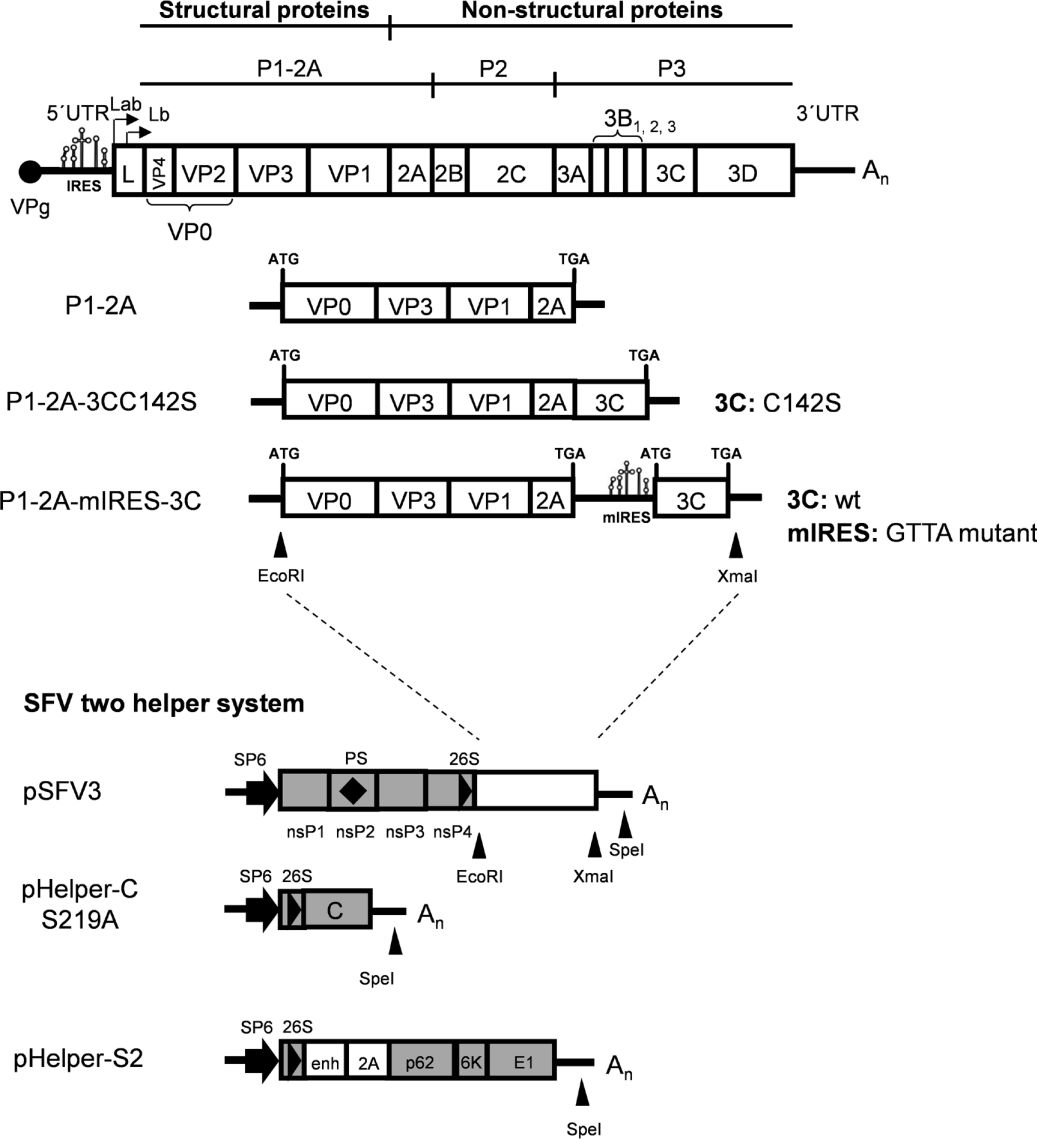
Schematic representation of the FMDV genome and the rSFV plasmids used in this study. The P1-2A, P1-2A-3CC142S and P1-2A-mIRES-3C FMDV cDNA cassettes have been described elsewhere [13,26] while the pSFV3 and the split helper plasmids have also been described previously [23]. Positions of relevant restriction enzyme sites used are shown. Abbreviations: P1-2A: capsid precursor protein; 3C: 3C^pro^ wild-type or C142S mutant; mIRES: internal ribosome entry site GTTA mutant; SP6: SP6 promotor; nsP1-P4: SFV non-structural proteins 1–4; PS: packaging signal; 26S: SFV 26S subgenomic promotor; C: SFV capsid; p62, 6K and E1: SFV spike proteins.

The FMDV cDNA cassettes used in this study, that encode the capsid precursor (P1-2A) alone or with 3C^pro^ are shown in Fig 1 and were prepared by standard methods [24]. The split-helper RNA system for production of single cycle rSFV virus particles was based on the use of the previously published pSFV3 [25], pSFV-helper-C-S219A and pSFV-helper-S2 vectors [23]. The cDNA sequences corresponding to the O1 Manisa FMDV capsid precursor (in pGEM-3Z-O-P1-2A) alone or together with a mutant form of 3C^pro^, with reduced catalytic activity (3CC142S from pGEM-3Z-O-P1-2A-3CC142S), or with low level expression of the wt 3C (IRESgtta3Cwt from pGEM-3Z-O-P1-2A-IRESgtta3Cwt) have been described elsewhere [13,26]. These three plasmids were digested with EcoRI and XmaI to release the FMDV cDNA cassettes, and the pSFV3 vector was digested with BglII and XmaI and separately with BglII and EcoRI (the pSFV3 vector contains three EcoRI sites). The plasmids pSFV3-FMDV-P1-2A, pSFV3-FMDV-P1-2A-3CC142S and pSFV3-FMDV-P1-2A-mIRES3Cwt (with the serotype O P1-2A-IRESgtta3Cwt cassette) were made through single-step three-part ligations (between the different EcoRI/XmaI fragments and the two parts of the backbone vector, i.e. a XmaI/BglII fragment and a BglII/EcoRI fragment). Plasmids were amplified in *Escherichia coli* (Top10, Invitrogen), purified (Midiprep kit, Fermentas) and verified by sequencing.

### *In vitro* transcription, electroporation and packaging of recombinant SFV-FMDV RNAs

The methods used to transcribe capped RNAs *in vitro*, electroporate the RNA into BHK (baby hamster kidney, ATCC-CCL-10) cells and to produce single-cycle infectious rSFV particles were performed as previously described [22]. Briefly, the plasmids were linearized by digestion with SpeI, purified (QIAquick PCR purification kit, Qiagen) and *in vitro* transcribed using SP6 RNA polymerase (mMessage mMachine kit, Ambion), in 20 μl reactions, as described by the manufacturer. The integrity of the RNA transcripts was analysed using agarose gel electrophoresis and the transcripts were introduced into BHK cells by electroporation, as described previously [27,28]. To package the recombinant RNAs into rSFV particles using the two-helper RNA system, 20 μl of each RNA transcription reaction was used (i.e. a particular rSFV RNA plus the two separate helper RNA transcripts). The medium containing the rSFV particles was harvested, following incubation at 33°C, within 48 h post electroporation, after the development of cytopathic effect (CPE), and clarified by centrifugation at 40,000xg for 30 min at 4°C. To concentrate and purify the rSFV-FMDV particles from the medium (when necessary, only for animal experiments 1 and 2), they were sedimented, by ultracentrifugation, through a 20% sucrose cushion at 140,000xg for 90 min at 4°C and resuspended in 50 mM Tris-HCl (pH 7.4), 100 mM NaCl and 0.5 mM EDTA. The rescued rSFVs were termed: rSFV-FMDV-P1-2A, rSFV-FMDV-P1-2A-3CC142S and rSFV-FMDV-P1-2A-mIRES-3C respectively.

### Immunofluorescence assays

Titres of the packaged rSFV-FMDV particles (as infectious rSFV-FMDV units/ml) were quantified by immunofluorescence assays essentially as described previously [22]. Briefly, monolayers of BHK cells, grown on glass coverslips in 35 mm wells plates, were infected with 10-fold dilutions of the harvested rSFV-FMDV particles. After incubation at 37°C for 10 to 16 h, the cells were fixed, stained and mounted as previously described [29]. In brief, to identify cells containing FMDV-capsid proteins, the cells were stained using a polyclonal rabbit anti-FMDV O1 Manisa antibody followed by a donkey Alexa-fluor 568-labelled anti-rabbit IgG (A10042, Life Technologies) and viewed using an epifluorescence microscope.

### Infection of cells with rSFV-FMDV particles

BHK, IBRS-2 (porcine kidney, ATCC CRL-1835) and pBTY (primary bovine thyroid, produced in-house) cells were infected with rSFV-FMDV particles at a multiplicity of infection (MOI) of 20 infectious units/cell for 1 h, as described previously [22], and incubated at 37°C. At 10 to 16 h post infection, cell lysates were prepared using 20 mM Tris-HCl (pH 8.0), 125 mM NaCl and 0.5% NP-40, and clarified by centrifugation at 18,000 x g for 10 min at 4°C. Samples were examined for the presence of FMDV proteins by immunoblotting and enzyme-linked immunosorbent assays (ELISA) as described below.

### Immunoblotting

Immunoblotting was performed according to standard methods as described previously [26]. Briefly, samples were mixed with Laemmli sample buffer (with 25 mM dithiothreitol), separated by SDS-PAGE (12.5% or 15% polyacrylamide) and transferred to polyvinylidene difluoride membranes (PVDF, Millipore). These were incubated with primary antibodies specific for the FMDV capsid proteins (anti-FMDV O1 Manisa guinea pig serum), FMDV 2A (ABS31, Millipore), actin (ab8227, Abcam) or FMDV 3C^pro^ (anti-FMDV 3C 1G1, kindly provided by E. Brocchi, Brescia, Italy, as used previously [30]. Immunoreactive proteins were visualized using appropriate secondary horseradish peroxidase-conjugated antibodies (Dako) and a chemiluminescence detection kit (ECL Prime, Amersham) with a Chemi-Doc XRS system (Bio-Rad).

### ELISAs

Serotype-specific FMDV antigen ELISAs (for serotype O) were performed as described previously [31,32]. The ELISA to detect FMDV antigen binding to the integrin α_v_β_6_ (a cellular receptor for FMDV) was performed as described [26].

### Sucrose gradient analyses

Cell extracts from rSFV-FMDV infected BHK cells (400 μl of lysate prepared from one 35 mm well per gradient) were loaded onto 10 to 30% (w/v) sucrose gradients and centrifuged as previously described [29]. Viral proteins were detected in collected fractions by the serotype-specific FMDV antigen ELISA (as described above).

### Ethics Statement

All animal work was approved and conducted according to the requirements of the Danish Animal Experiment Inspectorate (Licence nos. 2012-15-2934-00182 and 2012-15-0201-00173). Susceptible cattle exhibited typical clinical signs of FMD following virus challenge but all recovered in a few days and none died as a result of this infection. At the termination of the experiment, all animals were humanely euthanized.

### Vaccination and challenge of cattle

In experiment 1, eight cattle of 2–6 months of age were divided into three groups, one group with two animals (control group 1, animals C1 and C2) and the other two groups with three animals in each (group 2, animals C3, C4 and C5 and group 3, animals C6, C7 and C8). Group 2 (C3-C5) and group 3 (C6-C8) were vaccinated subcutaneously with 5x10^8^ infectious units of rSFV-FMDV-P1-2A or rSFV-FMDV-P1-2A-mIRES-3C that express the FMDV P1-2A or FMDV P1-2A-mIRES-3C cassettes respectively. The control group received injections of PBS. The procedure used to challenge the cattle has been described elsewhere [33]. Briefly, at post vaccination day (PVD) 21, the animals were challenged by sub-epithelial injection, in the tongue, with ca. 10^6^ TCID_50_ (in total, as determined in pBTY cells) of FMDV O UKG 34/2001. All animals were monitored daily, with measurements of rectal temperature and observation of clinical signs (drooling and appearance of lesions in the mouth and on the feet). Serum samples were collected at predetermined times until PVD 30 when the experiment was terminated.

In experiment 2, thirteen calves were divided into 5 groups. The control groups, 1 (C1, C2) and 2 (C3, C4), each consisted of two cattle while the test groups 3 (C5-C7), 4 (C8-C10) and 5 (C11-C13) each comprised 3 animals. Cattle in groups 3, 4 and 5 each received 7.5 x 10^8^ infectious units of the rSFV-FMDV-P1-2A-mIRES-3C on PVD 0 and then again on PVD 14. Animals in groups 1 and 3 were inoculated into the tongue with FMDV O UKG 34/2001 (ca. 10^6^ TCID_50_) on PVD 28. Animals in group 2 (C3-C4, unvaccinated) and group 5 (C11-C13, twice vaccinated) were kept in close contact with those from group 1 (C1-C2) from one day after challenge (PVD 29) in one stable while cattle in group 4 (C8-C10) were kept in contact with the vaccinated and inoculated cattle (C5-C7) in group 3 within another stable. All animals were monitored daily and blood samples were collected at pre-determined times until day 42 when the experiment was terminated.

In the animal experiment 3, nine calves were divided equally into 3 groups. The control group 1 (C1-C3) was unvaccinated while the group 2 (C4-C6) was inoculated with rSFV-FMDV-P1-2A-mIRES-3C (4 x 10^8^ infectious units) on PVD 0 and then boosted on PVD 14 with ca. 10 μg of O1 Manisa empty capsid particles with Montanide ISA 201 VG (Seppic) mineral oil adjuvant. Group 3 (C7-C9) received the same inoculations but in the opposite order, thus the animals were inoculated with the O1 Manisa empty capsid particles (in adjuvant) on PVD 0 and then with the rSFV-FMDV-P1-2A-mIRES-3C on PVD 14. The empty capsid particles were produced by dual infection of RK13 (rabbit kidney, ATCC CCL-37) cells with the vaccinia virus vTF7-3 [34], that expresses the T7 RNA polymerase, and another vaccinia virus containing a T7-P1-2A-3C^pro^ O1 Manisa cDNA cassette [35]. The empty capsids were then purified by sucrose gradient centrifugation essentially as previously described [11]. All animals were challenged on PVD 28 by needle inoculation into the tongue (as in experiment 1, see above).

The presence of FMDV RNA and anti-FMDV antibodies in the cattle serum samples were determined by RT-qPCR (targeting the 5'-UTR [36]) and a blocking ELISA [37], respectively. The level of viral RNA detected in serum samples was converted to the number of genome copies by reference to a standard curve of reference RNA samples, assayed in parallel, as described previously [27]. Selected serum samples were titrated in the blocking ELISA, using 2-fold dilutions from an initial 1:5 dilution. The titre is the lowest dilution giving a positive signal.

The presence of neutralising anti-FMDV antibodies in sera was determined using virus neutralization assays (VNTs) with O1 Manisa FMDV (ca. 100 TCID_50_) in IBRS2 cells, essentially as described previously [31]. Each serum dilution was assayed in 4 wells and the appearance of CPE was scored following incubation for 72 hrs. Results were expressed as reciprocal VNT titres derived from the serum dilution giving 50% neutralization and are plotted as log_2_ values.

## Results

### Construction of pSFV-FMDV plasmids

In previous studies [13,26], we have described the three serotype O FMDV cDNA cassettes indicated in Fig 1. One cassette encodes the FMDV capsid precursor P1-2A (from O1 Manisa) alone while the P1-2A-3CC142S cassette encodes the FMDV P1-2A linked directly (within a single open reading frame (ORF)) to a mutant form of the FMDV 3C^pro^ with much reduced catalytic activity [38]. The third FMDV cDNA cassette, P1-2A-mIRES-3C, includes two separate ORFs for P1-2A and the wt 3C^pro^; the expression of the 3C^pro^ is dependent on a mutant form of the FMDV IRES element (termed GTTA) that is highly defective (ca. 10% of wt activity) and therefore only produces a relatively low level of the protease relative to the capsid precursor. Each of these three cassettes has been introduced into the SFV expression vector called pSFV3 [23] so that the production of the RNA transcripts containing the FMDV sequences is dependent on the activity of the SFV 26S sub-genomic promoter (Fig 1).

### Expression of FMDV capsid proteins using the SFV two-helper system

**Fig 2:**
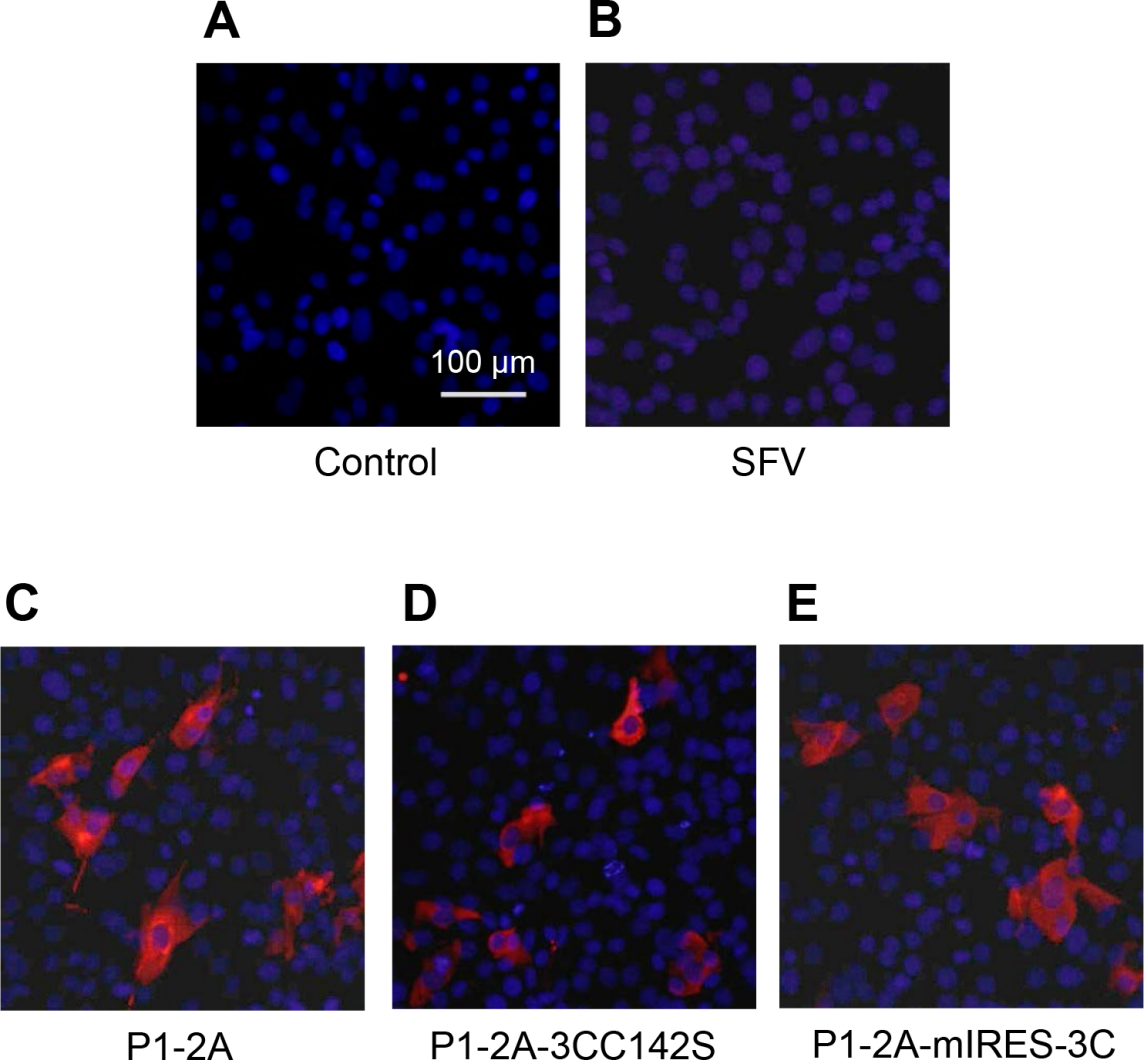
Expression of FMDV capsid proteins using the SFV split-helper system. Uninfected BHK cells (A) or cells infected with rSFV (B), rSFV-FMDV-P1-2A (C), rSFV-FMDV-P1-2A-3CC142S (D) or rSFV-FMDV-P1-2A-mIRES-3C (E), at an MOI of 20, were immunostained at 16 h post infection. FMDV proteins were detected with an anti-FMDV O1 Manisa polyclonal antibody and a secondary antibody labeled with Alexa Fluor 568 (red). The cellular nuclei were visualized with DAPI (blue). Bar, 100 μm. The results shown are representative of three independent experiments.

Capped RNA transcripts were prepared *in vitro*, using SP6 RNA polymerase, from pSFV3 and its 3 derivatives containing the FMDV cDNA cassettes and also from the two helper plasmids pHelper-CS219A and pHelper-S2 (see Fig 1). The production and integrity of the RNA transcripts was verified by agarose gel electrophoresis and then each rSFV transcript was electroporated into BHK cells in conjunction with the two helper RNAs. Following incubation at 33°C, CPE was observed and virus harvests were prepared; these were termed rSFV3 (no FMDV insert), rSFV-FMDV-P1-2A, rSFV-FMDV-P1-2A-3CC142S and rSFV-FMDV-P1-2A-mIRES-3C. To titrate these virus stocks, known dilutions were used to infect BHK cells and after 16 h the cells were fixed and stained for the presence of FMDV capsid proteins (data not shown). The expression of FMDV proteins in BHK cells infected with each of these virus stocks is shown in Fig 2. As expected, no staining for FMDV proteins was observed in mock or rSFV3-infected cells (Fig 2A and 2B). In contrast, FMDV proteins were detected from each of the rSFV-FMDVs that express sub-genomic RNA transcripts including the FMDV sequences (Fig 2C–2E).

### Characterization of expressed FMDV capsid proteins

**Fig 3:**
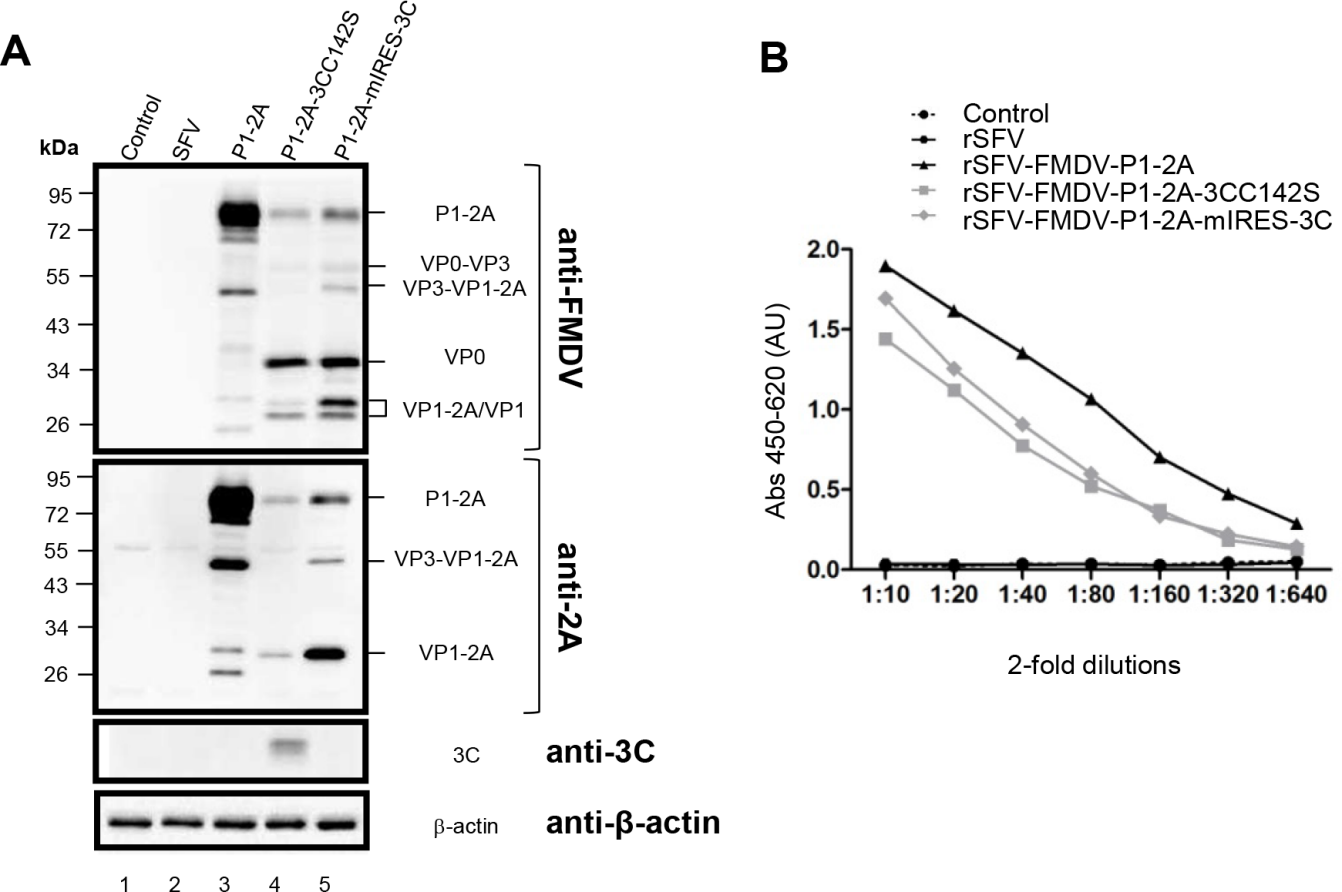
Characterization of expressed FMDV capsid proteins. (A) Uninfected BHK cells (control, lane 1) or cells infected with rSFV (lane 2), rSFV-FMDV-P1-2A (lane 3), rSFV-FMDV-P1-2A-3CC142S (lane 4) or rSFV-FMDV-P1-2A-mIRES-3C (lane 5) were harvested and the cell lysates were fractionated by SDS–PAGE. The proteins were transferred to PVDF membrane and probed with antibodies specific for FMDV capsid proteins (top), FMDV 2A (second), FMDV 3C^pro^ (third) and β-actin (bottom) as indicated. Detection of β-actin was used as a control for equal protein loading. The results shown are representative of three independent experiments. Molecular mass markers (kDa) are indicated on the left. (B) Cell lysates, as used in panel A, were diluted (10-fold initially and then 2-fold dilutions) and analysed with a FMDV serotype O-specific antigen ELISAs. The results shown are representative of two independent experiments. AU, absorbance units.

In order to confirm that the expected FMDV proteins were being expressed within cells infected with the different rSFVs, cell lysates were prepared and analysed by immunoblotting using antibodies specific for the FMDV capsid proteins, for the FMDV 2A and also for the FMDV 3C^pro^ (Fig 3A). No products were detected by these antibodies in mock or rSFV3 infected cells (lanes 1–2). However, as expected, the rSFV-FMDV-P1-2A expressed the intact capsid precursor (apparent Mr ca. 90kDa, see lane 3) and processing to the mature capsid proteins (including VP0 and VP1) was observed from the rSFV-FMDVs that also encoded the FMDV 3C^pro^ (see lanes 4 and 5; note, the VP3 is not detected by this anti-FMDV antiserum). Consistent with earlier studies using the vaccinia virus transient expression system [13], processing of the VP1/2A junction was incomplete when low levels of the 3C^pro^ activity were produced (Fig 3A, lanes 4 and 5); note the presence of VP1-2A, as detected by the anti-2A antiserum. It appears that processing of the VP1/2A junction is the slowest of the 3C^pro^-mediated cleavages within the P1-2A precursor in cells. The 3C^pro^ itself, could only be detected in the immunoblot from the rSFV-FMDV-P1-2A-3CC142S virus (Fig 3A, lane 4). Due to the low level of protease activity of this mutant protein, the expression systems are able to tolerate higher levels of this protein than wt 3C^pro^. However, clearly the level of 3C^pro^ activity achieved by the low-level expression of the wild-type protease from the P1-2A-mIRES-3C cassette was sufficient to achieve efficient processing of the P1-2A (Fig 3A, lane 5).

To determine the antigenicity of the FMDV proteins expressed by the rSFV-FMDV vectors, samples of the infected cell lysates were also tested in a FMDV serotype specific antigen ELISA. No signal was detected from the mock-infected or control rSFV3 virus infected BHK cells (Fig 3B) but strong signals were observed with each of rSFV-FMDV infected-cell lysates, i.e. including both the intact and processed forms of P1-2A, consistent with previous results using the vaccinia virus expression system [13]. Furthermore, similar results were obtained using the various rSFV vectors in pBTY (bovine) and IBRS-2 (porcine) cells (see S1 Fig).

### Assembly of FMDV empty capsids expressed by rSFV-FMDVs

**Fig 4:**
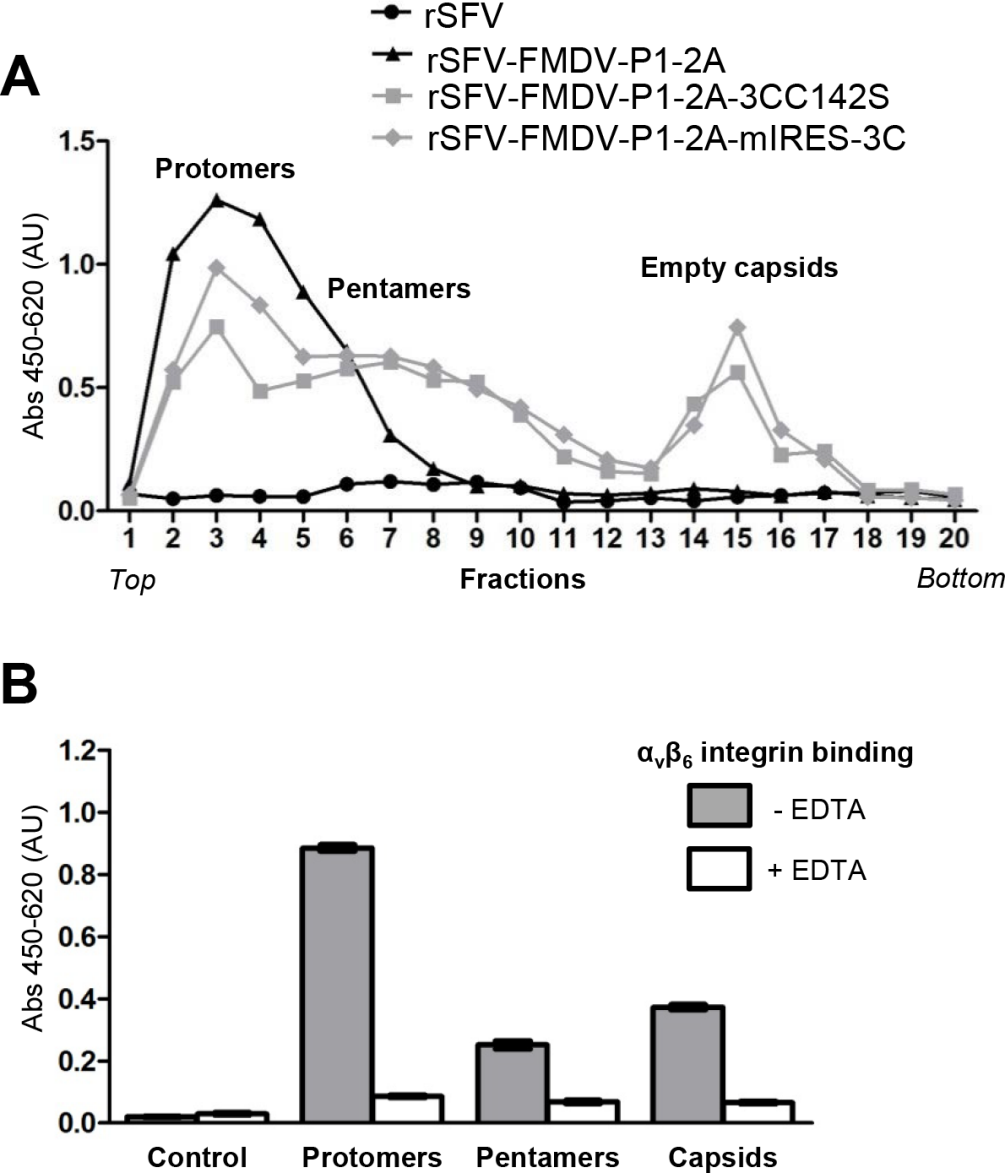
Assembly and properties of FMDV protomers, pentamers and empty capsids expressed from rSFV vectors. (A) BHK cells were infected as described in Fig 2 using the indicated rSFV-FMDVs. Cytoplasmic extracts were prepared (16 h post infection) and sedimented through sucrose gradients (10–30%) and fractionated. FMDV proteins from each fraction were detected using a serotype-specific antigen ELISA. The location of protomers, pentamers and empty capsids are indicated. (B) Fractions (fr) from the rSFV-FMDV-P1-2A-mIRES-3C infected BHK cells containing protomers (fr. 3), pentamers (fr. 7) and empty capsids (fr. 15) were assayed to detect FMDV antigen binding, in the presence or absence of EDTA as indicated, to the integrin α_v_β_6_ coated directly onto plates. Binding buffer was used as the control. Results are presented as mean ± SEM of triplicate samples.

The processed products of P1-2A can assemble into empty capsid particles (which sediment at ca. 80S) and have much higher immunogenicity than the unprocessed P1-2A or unassembled pentamers [7,39,40]. In order to assess the assembly of the products expressed from the rSFV-FMDVs, infected cell lysates were analysed on sucrose gradients and the presence of FMDV proteins, as detected by ELISA, in each gradient fraction was determined (Fig 4A). The intact P1-2A remains close to the top of the gradient (mainly fractions 2–5) whereas the 3C^pro^ processed products (expressed from the P1-2A3CC142S and P1-2A-mIRES-3C cassettes) migrate much further into the gradient (in fractions 14 and 15) consistent with assembly into empty capsid particles. Processed proteins that assembled into pentamers, an assembly intermediate, migrate in an intermediate position (around fraction 7).

The ability of the FMDV capsid proteins to bind specifically to the integrin α_v_β_6_ (a cellular receptor for FMDV) was also assessed using an ELISA (Fig 4B). The protomers, pentamers and empty capsids were each able to bind specifically to this integrin in a divalent cation dependent manner (binding was blocked in the presence of EDTA).

### Vaccination of cattle with rSFV-FMDVs (animal experiment 1)

**Fig 5:**
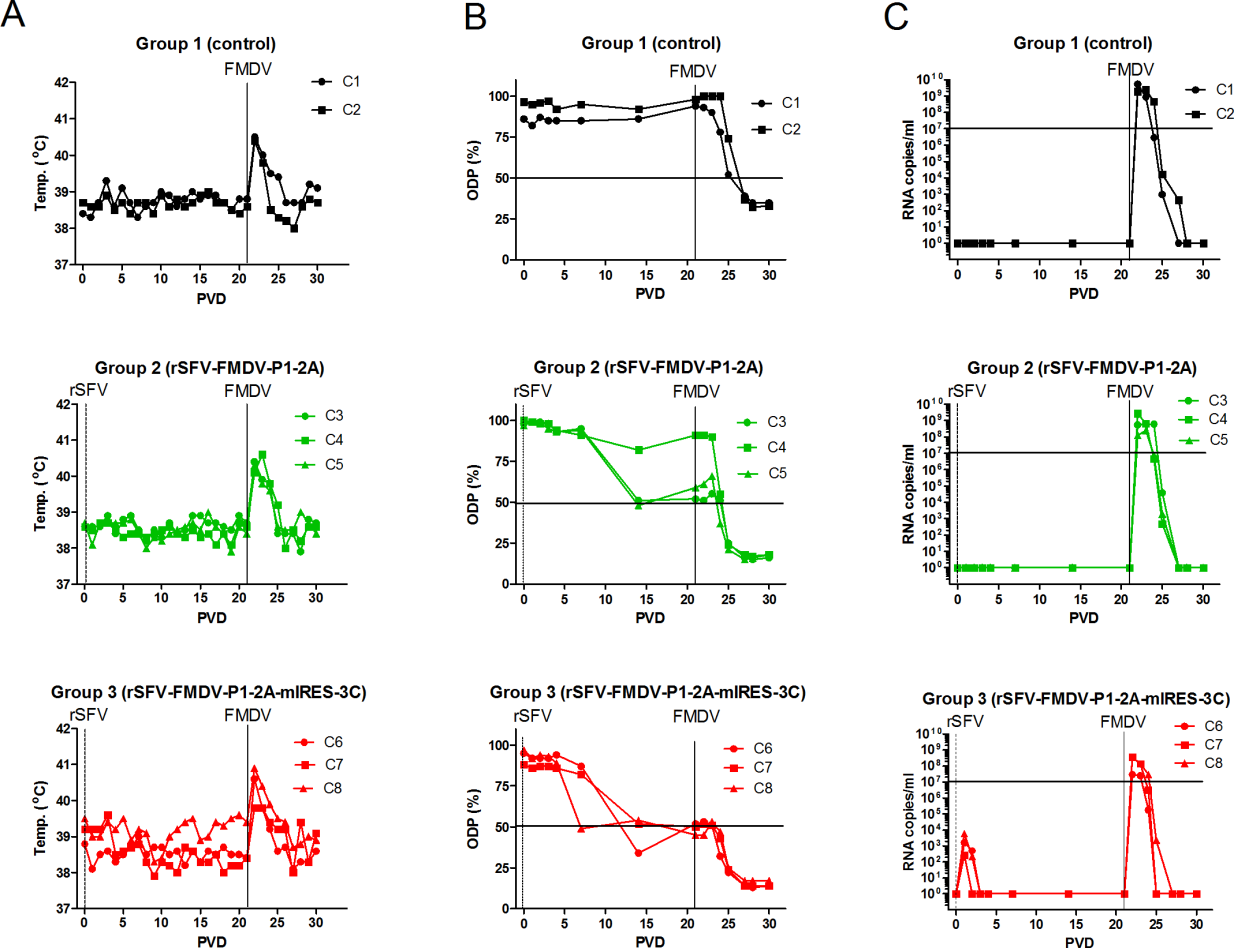
Vaccination of cattle with rSFV-FMDV particles and response to FMDV challenge. The indicated animals (in experiment 1) were unvaccinated (controls, C1, C2) or vaccinated on post-vaccination day (PVD) 0 with either the rSFV-FMDV-P1-2A (calves C3-C5) or the rSFV-FMDV-P1-2A-mIRES-3C (calves C6-C8) vectors (indicated by vertical dotted line, marked rSFV) and challenged with FMDV on PVD 21 (indicated by vertical line, marked FMDV). (A) Body (rectal) temperatures in each animal were monitored on a daily basis. (B) Serum samples collected at each indicated day were assayed for anti-FMDV antibodies in a serotype O-specific blocking ELISA. The diagnostic cut-off level (50%) in the ELISA is indicated by the horizontal line. (C) The number of FMDV RNA copies present in the serum was determined by RT-qPCRs by reference to a dilution series of a known concentration of FMDV RNA. The results are presented as copies of FMDV RNA/ml of serum. A level of 10^7^ copies/ml is indicated by a horizontal line.

As an initial assessment of the ability of the single-cycle rSFV-FMDV vectors to induce a protective anti-FMDV response in a natural host, cattle were vaccinated with ca. 5 x 10^8^ infectious doses of either the rSFV-FMDV-P1-2A (group 2) or rSFV-FMDV-P1-2A-mIRES-3C (group 3) or mock-vaccinated with PBS (group 1). After 21 days, the animals were challenged with FMDV O1/UKG 2001 (ca. 10^6^ TCID_50_) by tongue inoculation. The vaccination with the rSFV-FMDVs induced no change in body temperature and it was only after challenge with FMDV that fever was induced (especially on PVD 22) (Fig 5A). There was no apparent difference in the appearance of clinical signs of FMD in the control animals (group 1) or the vaccinated animals. All showed typical signs of FMDV infection including elevated body temperature (Fig 5A), excess salivation and mouth lesions away from the site of inoculation (see fuller description of clinical signs associated with FMDV infection below).

Although protection against FMDV challenge was not achieved in this initial experiment, analysis of the sera (by ELISA) indicated that an anti-FMDV response was induced in the animals that received the rSFV-FMDV-P1-2A and the rSFV-FMDV-P1-2A-mIRES-3C. These responses appeared strongest at PVD 14 but then either remained constant or declined (Fig 5B). Following challenge on PVD 21, there was a boost in the anti-FMDV response, as measured by the ELISA, that was apparent from PVD 24 or 25 in all animals and this was maintained until termination of the experiment on PVD 30. The unvaccinated animals also seroconverted against FMDV by PVD 27 following challenge. Titration of the sera (initially screened at 1:5 dilution) indicated that the level of anti-FMDV antibodies induced by the rSFV-FMDVs alone was fairly modest (only up to a titre of 1:10). However, the initial vaccination with the rSFV-FMDVs strongly enhanced the level of anti-FMDV antibodies (titres of 1:320 and above) that could be detected, post-challenge, on PVD 27 and 30 (see Table 1). This suggested that these vectors had primed the anti-FMDV immune response. In contrast, in the unvaccinated animals, the titre of anti-FMDV antibodies was only 1:20 post-challenge (Table 1).

**Table 1:** Reciprocal titres of anti-FMDV antibodies (serotype O) in sera from unvaccinated and rSFV-FMDV vaccinated calves in experiment 1.

			Pre-challenge		Post-challenge	
Group	Vaccination and challenge	Animal	PVD 14	PVD 21	PVD 27	PVD 30
1	No vaccination (control)	C1	-	-	-	20
	Needle challenge	C2	-	-	-	20
2	rSFV-FMDV-P1-2A	C3	-	-	320	320
	Needle challenge	C4	-	-	320	320
		C5	-	5	640	320
3	rSFV-FMDV-P1-2A-mIRES-3C	C6	5	10	320	320
	Needle challenge	C7				
		C8				

Calves C3 to C8 were vaccinated with the indicated rSFV-FMDV on PVD 0 and then challenged with FMDV by needle inoculation on PVD 21. To determine the titre, sera collected from calves on PVD 14, 21, 27 and 30 were assayed in the ELISA using 2-fold dilutions starting at 1:5.—indicates negative.

Detection of FMDV RNA in the cattle sera by RT-qPCR (Fig 5C), identified the presence of the FMDV derived RNA on PVD 1 circulating in all 3 inoculated animals in group 3 (this was maintained in 2 of the animals on PVD 2 as well). The assay detects sequences within the FMDV IRES that is present within the rSFV-FMDV-P1-2A-mIRES-3C particles and thus indicates that short term, low level (ca. 10^3^ copies/ml), circulation of the packaged rSFV-FMDV RNA occurred following vaccination. Since no IRES sequences are present in the rSFV-FMDV-P1-2A then no signal for FMDV RNA could be expected to be observed in the sera from group 2 prior to challenge and this was indeed the result (Fig 5C). The presence of high levels of FMDV RNA was detected in the serum of all animals following tongue inoculation with the infectious FMDV on PVD 21. Viremia was apparent on PVD 22–24 in group 3 and from PVD 22 to PVD 25 in group 2 and extended through to PVD 27 in the control animals in group 1 (Fig 5C). The highest levels of viral RNA were observed in the sera from control animals (up to 5.3 x10^9^ RNA copies/ml in calf C1 and 2.4 x 10^9^ copies/ml in calf C2) while the peak level of viremia in animals that had received the rSFV-FMDV-P1-2A-mIRES-3C were rather lower. In calf C6, the peak level of FMDV RNA in serum was ca. 2.9 x 10^7^ RNA copies/ml while 4 x 10^8^ copies/ml observed in calves C7 and C8 (calves C6-C8 are each in group 3). The profile of viremia in group 2 (that received the rSFV-FMDV-P1-2A) was very similar to the control animals. With this small sample size, the significance of these differences in viremia (ca.10-100-fold reduction in peak levels) in the group 3 animals is not certain but a trend of shorter duration and a lower level of viremia in the rSFV-FMDV-P1-2A-mIRES-3C vaccinated cattle seems consistent.

### Booster vaccination and contact challenge (animal experiment 2)

In an attempt to enhance the level of anti-FMDV antibodies prior to challenge, a second experiment was performed in which a second vaccination with the rSFV-FMDV was given prior to exposure of the animals to FMDV. In addition, two alternative challenge routes were used; these were either direct needle inoculation of virus into the tongue, as in the first experiment, or a contact challenge by exposure to FMDV infected calves. In this experiment, calves C1-C4 were unvaccinated while calves C5-C13 were each vaccinated with the rSFV-FMDV-P1-2A-mIRES-3C (7.5 x 10^8^ infectious units) on PVD 0 and then again on PVD 14. On PVD 28, the control calves C1 and C2 plus the vaccinated calves C5-C7 were challenged with FMDV O UKG 34/2001 (ca. 10^6^ TCID_50_) into the tongue (as above). The inoculated calves C1 and C2 were then kept in contact with the naïve calves C3 and C4 plus the vaccinated calves C11 to C13 in one stable (to determine if transmission could occur). Meanwhile, the calves C5 to C7 were kept in contact with calves C8 to C10 in a separate stable to assess whether transmission of FMDV from the vaccinated and then needle-challenged animals to vaccinated animals occurred.

The body temperatures of all cattle remained very constant (following primary and secondary vaccination) until after challenge with FMDV (representative data is shown in the S2 Fig, panels A and B). In the animals that were directly challenged with FMDV on PVD 28, fever became apparent in both the control (C1, C2) and vaccinated animals (C5-C7) on PVD 29 and 30 (S2 Fig, panels A and B). Fever was also observed (during the period PVD 31 to 36) in the animals kept in contact (from PVD 29) with the challenged animals (data not shown). Typical clinical signs of FMD were also observed during this period in all animals as in experiment 1 (data not shown).

In the blocking ELISA (using 1:5 dilution of sera, see S2 Fig, panels C and D), it was apparent that animals (C5-C7) which received the rSFV-FMDV-P1-2A-mIRES-3C vector seroconverted against FMDV (by 7 or 14 days when next sampled) following the primary vaccination. There was no apparent increase in the response following the second vaccination on PVD 14 but the presence of anti-FMDV antibodies was maintained. Following challenge with FMDV, the level of anti-FMDV antibody increased to some degree in both the FMDV inoculated animals (C5 to C7 by PVD 34, see S2 Fig, panel D) and in the contact challenged animals (C8-C13 by PVD 37). However, these assays are only semi-quantitative and the results of the titrations are more informative (see below). In the non-vaccinated animals, no anti-FMDV antibodies could be detected prior to challenge but the FMDV-inoculated calves seroconverted by PVD 34 (S2 Fig, panel C) while the in-contact animals seroconverted by PVD 37.

Selected sera, based on the results from the screening ELISA, were titrated (see Table 2). At PVD 14, fairly low antibody titres (1:5 to 1:40) were present in 7 of the 9 animals vaccinated with the rSFV-FMDV-P1-2A-mIRES-3C and by PVD 28 (14 days after the second vaccination) all 9 animals were seropositive but the antibody titres were still in the range of 1:5 to 1:40. Following FMDV challenge, on PVD 28 (by inoculation) or PVD 29 (by contact), there was a large rise in the level of antibodies to FMDV in the vaccinated animals (frequently to titres of 1:1280 or higher, see Table 2). In contrast, in the non-vaccinated animals (groups 1 and 2), the post-challenge titres only reached 1:20 or 1:40. Thus, as in experiment 1, the inoculation with the rSFV-FMDV-P1-2A-mIRES-3C strongly primed the anti-FMDV antibody response that was generated post-challenge.

**Table 2:** Reciprocal titres of anti-FMDV antibodies (serotype O) in sera from unvaccinated and rSFV-FMDV vaccinated calves in experiment 2.

			Pre-challenge		Post-challenge
Group	Vaccination and challenge	Animal	PVD 14	PVD 28	PVD 42
1	No vaccination (control)	C1	-	-	40
	Needle challenge	C2	-	-	40
2	No vaccination (control)	C3	-	-	20
	Contact challenge from group 1	C4	-	-	20
3	rSFV-FMDV-P1-2A-mIRES-3C x 2	C5	5	20	1280
	Needle challenge	C6	20	40	2560
		C7	40	20	640
4	rSFV-FMDV-P1-2A-mIRES-3C x 2	C8	-	5	640
	Contact challenge from group 3	C9	-	5	320
		C10	20	40	1280
5	rSFV-FMDV-P1-2A-mIRES-3C x 2	C11	40	20	2560
	Contact challenge from group 1	C12	5	5	2560
		C13	5	5	1280

Calves C5 to C13 were vaccinated with rSFV-P1-2A-mIRES-3C on PVD 0 and also on PVD 14 and then challenged with FMDV by needle inoculation on PVD 28 (groups 1 and 3) or by contact with inoculated calves from PVD 29 (groups 2, 4 and 5, see text). To determine the antibody titres, sera collected from calves on PVD 14, 28 and 42 were assayed in the ELISA using 2-fold dilutions starting at 1:5.—indicates negative.

The presence of FMDV RNA in the cattle serum was determined by RT-qPCR. As in the first experiment, the primary vaccinations of the cattle with the rSFV-FMDV-P1-2A-mIRES-3C resulted in a weak signal, corresponding to the presence of the FMDV IRES in this recombinant virus, on PVD 1 and, in some cases on PVD 2 (see S2 Fig, panel F). However, no such signal was detected in the sera following the second vaccination, with the same material, on PVD14. Following challenge with FMDV, a large increase in viremia (as judged by the level of FMDV RNA in the serum) was apparent in all animals but, consistent with the first experiment, the level of viremia was greatly reduced (up to 1000-fold) in the vaccinated animals compared to the control animals (S2 Fig, panels E and F). It is apparent that the vaccinated and then FMDV-challenged animals (group 3) were still able to transmit the virus to other vaccinated animals (group 4, see Table 2) as they became infected. Indeed, all animals showed typical clinical disease following challenge.

### A prime-boost vaccination strategy prevents FMDV circulation and disease (animal experiment 3)

To determine if the use of a prime-boost strategy, using two different types of vaccine would be effective in combating virus replication, a third experiment was performed in which the combination of the rSFV-FMDV single-cycle virus infection and administration of purified recombinant FMDV empty capsid particles was tested. Animals in group 1 (C1-C3) were unvaccinated. Animals in group 2 (C4-C6) were vaccinated with rSFV-FMDV-P1-2A-mIRES-3C on PVD 0 and then boosted with FMDV empty capsids on PVD 14. The calves in group 3 (C7-C9) were given the same components but in the reverse order. All the animals in each group were challenged with FMDV on PVD 28 by needle inoculation.

#### a) Clinical signs of FMDV-infection post-challenge

**Fig 6:**
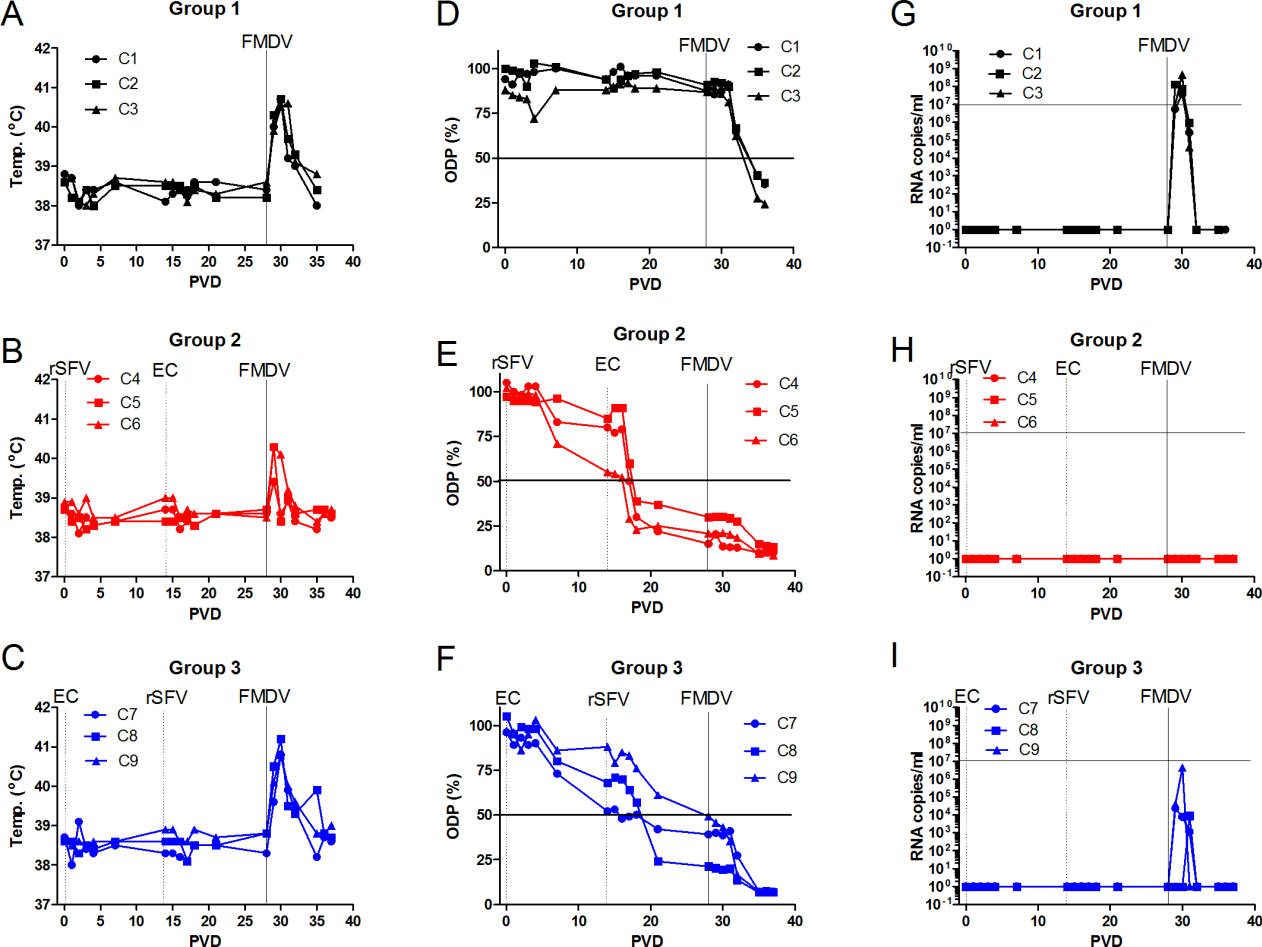
Prime-boost vaccination strategy effectively induces anti-FMDV antibodies in cattle and blocks virus circulation post-challenge. In experiment 3, calves in group 1 (panels A, D, G) were unvaccinated. Calves in group 2 (panels B, E, H) were vaccinated with rSFV-FMDV-P1-2A-mIRES-3C on PVD 0 (dotted vertical line, marked rSFV) and then with O1 Manisa empty capsid particles on PVD 14 (dotted vertical line, marked ECs). Calves in group 3 (panels C, F, I) were vaccinated with O1 Manisa empty capsid particles on PVD 0 (dotted vertical line, marked ECs) and then with rSFV-FMDV-P1-2A-mIRES-3C on PVD 14 (dotted vertical line, marked rSFV). All animals were challenged with FMDV (O UKG/34/2001) on PVD 28 (indicated by solid vertical line, marked FMDV). Body temperatures in each animal were monitored on a daily basis (panels A-C). Sera were collected from each calf on the indicated days and assayed in the serotype O blocking ELISA (panels D-F). Results are presented as ODP (%) with a cut-off value of 50% (indicated by horizontal line). The presence of FMDV RNA circulating in serum was assayed by RT-qPCR (panels G-I). The results are presented as copies of FMDV RNA/ml of serum as in Fig 5. A level of 10^7^ copies/ml is indicated by a horizontal line.

No body temperature changes were observed during the first 28 days of the experiment, prior to challenge (Fig 6A–6C). However, the calves in groups 1 and 3 showed a marked body temperature increase for at least 3 days post challenge with FMDV (Fig 6A and 6C) but only a small temperature response was detected in each of the animals in group 2 (Fig 6B) indicative of some local response at the site of virus inoculation.

In the unvaccinated group (control), all 3 animals (C1- C3) showed clinical signs consistent with FMDV infection after challenge. The day after needle challenge (PVD 29), the animals showed excess salivation and were reluctant to eat food but no lesions were observable by inspection of the mouth cavity. On the following day (PVD 30), lesions at the inoculation sites were visible. Furthermore, at PVD 31 and 32, several lesions were observed in the mouth cavity and included secondary sites on the tongue, the dorsal palate and the gingiva. At PVD 33, the animals showed increased interest in food again and at PVD 35 all lesions had started healing. In general, excess salivation and decreased appetite were the most consistent clinical signs during the experimental period after FMDV challenge.

In group 2, each of the calves, C4-C6, that had been vaccinated with the rSFV-FMDV-P1-2A-mIRES-3C and then boosted with the empty FMDV capsid particles, showed some salivation from PVD 29 (visually to a lesser degree than calves in the control group), lesions were observed on the tongues at the FMDV inoculation sites from PVD30. However, no other lesions were seen. From PVD 32, the lesions at the sites of inoculation began to heal and by the end of the experiment the epithelia on the tongues of these cattle were intact. In general, sparse salivation and normal appetite were observed during the experimental period after FMDV challenge.

All 3 animals (C7-C9) in group 3 (which received the empty capsids followed by the rSFV-FMDV-P1-2A-mIRES-3C) showed increased salivation from PVD 29. Lesions were observed at the inoculation sites from PVD 30 and one animal had an additional single lesion in the mouth cavity behind the front teeth at this time. At PVD 31–32, extreme salivation was recorded as well as several lesions in the oral cavity on the tongue and dorsal palate in the calves. Furthermore, one calf had lesions on one foot (right hind limb) and one calf had a single lesion in the left nostril. At PVD 34, the calves had improved in their general condition and showed increased interest in food again. At PVD 35–36, the lesions had started to heal. In general, massive salivation and decreased appetite (as for calves in group 1) were observed during the experimental period after FMDV challenge.

At necropsy, after the termination of the experiment, healing lesions were observed on the feet and around the mouth in groups 1 and 3. In group 2, a healing lesion at the base of the tongue was observed in one animal only.

#### b) Induction of anti-FMDV antibodies

As expected, no anti-FMDV antibodies were detected in unvaccinated cattle (C1-C3) prior to challenge (Fig 6D). For calves C4-C6 (group 2), initial vaccination (on PVD 0) with the rSFV-FMDV-P1-2A-mIRES-3C particles was followed on PVD 14 with the serotype O Manisa empty capsid particles (produced by a vaccinia virus expression system). This antigen strongly boosted the anti-FMDV immune response detected at PVD 21 and PVD 28 (Fig 6E) compared to that seen at PVD 14 and produced an antibody titre of 1:40 to 1:320 (by ELISA) in all 3 animals (Table 3) at PVD 28. In group 3, comprising calves C7-C9, initial vaccination with the empty capsid particles produced only a relatively weak anti-FMDV response by PVD 14 but this was boosted, as determined at PVD 28, by the inoculation with the rSFV-FMDV-P1-2A-mIRES-3C but not to the same degree as in group 2 (see Fig 6F and Table 3).

**Table 3:** Reciprocal titres of anti-FMDV antibodies (serotype O) in sera from calves in experiment 3.

			Post-prime	Post-boost	Post-challenge
Group	Vaccination	Animal	PVD 14	PVD 28	PVD 36
1	No vaccination (control)	C1			
		C2			
		C3			
2	rSFV-FMDV-P1-2A-mIRES-3C	C4			
	(PVD 0) + empty capsid particles	C5			
	(PVD 14)	C6			
3	empty capsid particles (PVD 0) +	C7			
	rSFV-FMDV-P1-2A-mIRES-3C	C8			
	(PVD 14)	C9			

Calves were either unvaccinated (group 1) or vaccinated with rSFV-FMDV-P1-2A-mIRES-3C (on PVD 0) followed by empty capsid particles (on PVD 14) (group 2) or vaccinated with empty capsids on PVD 0 and then with rSFV-FMDV-P1-2A-mIRES-3C on PVD 14 (group 3). All cattle were challenged with FMDV by needle inoculation on PVD 28. Sera collected on PVD 14, 28 (pre-challenge) and 36 were titred in the blocking ELISA using 2-fold dilutions starting at 1:5.—indicates negative.

Following challenge with FMDV on PVD 28 there was only a moderate change in the level of anti-FMDV antibodies in the animals in group 2 (Fig 6E and Table 3) to 1:320 or 1:1280. There was a much stronger increase in the anti-FMDV immune response in group 3 (Fig 6F), compared to group 2, following challenge (Table 3) with titres of 1:5120 reached in each animal. The control animals also seroconverted against FMDV following challenge (Fig 6D) but the titres of antibodies were much lower (1:40 or 1:160) (Table 3).

#### c) Post-challenge viremia

In the control animals (C1-C3), high levels of viremia (as determined by RT-qPCR) could be detected in the sera following FMDV challenge (see Fig 6G) which lasted for 3 days, this was largely coincident with the elevated temperature response (Fig 6A). In contrast, there was a complete block on viremia in group 2 post challenge; no FMDV RNA could be detected in the sera from calves C4-C6 following inoculation with FMDV (Fig 6H). The complete lack of viremia was consistent with the absence of disease in the animals in this group. In group 3, a marked viremia was observed in one animal (C9), which lasted for 2 days (Fig 6I). In addition, a much lower level of FMDV RNA was detected in the serum of calf C7 for a period of 3 days. In the third calf (C8), only a very low viremia was detected, on a single day; this result is consistent with the fact that this calf had the highest level of anti-FMDV antibodies, within this group (see Fig 6F, Table 3), on PVD 28 (pre-challenge).

#### d) Induction of neutralizing antibodies

**Fig 7:**
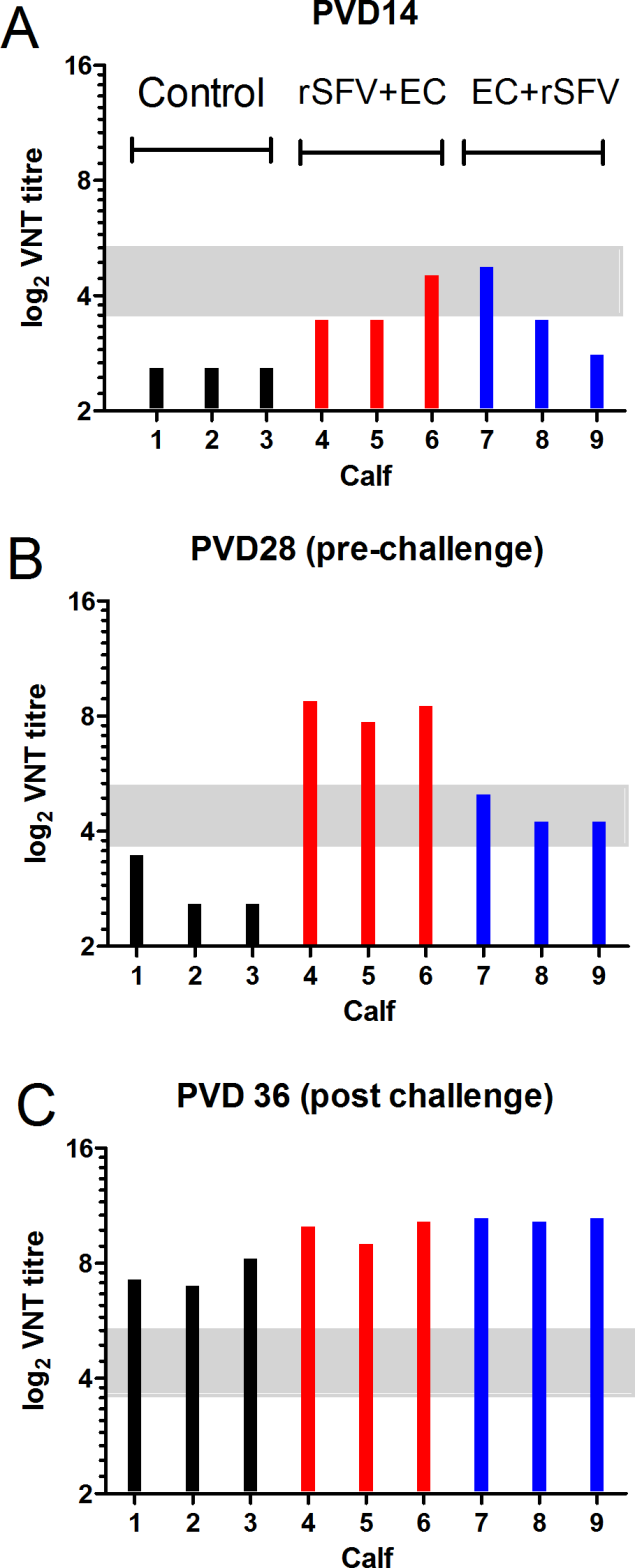
Production of neutralizing anti-FMDV antibodies in cattle. Sera from the calves in group 1 (C1-C3, black bars, control), group 2 (C4-C6, red bars, vaccinated with rSFV-FMDV-P1-2A-mIRES-3C on PVD 0 followed by empty capsids on PVD 14) and group 3 (C7-C9, blue bars, vaccinated with empty capsids on PVD 0 followed by rSFV-FMDV-P1-2A-mIRES-3C on PVD 14) were collected during the experiment 3. All animals were challenged with FMDV on PVD 28. Samples from PVD 14 (A), PVD 28 (B) (prior to challenge) and PVD 36 (C) (post challenge) were assayed for the presence of neutralizing anti-FMDV antibodies using VNTs. Results were calculated as reciprocal VNT titres and are displayed as log_2_ values. VNT titres ≤ 11 (log_2_ 11 = 3.459) are considered negative while titres ≥45 (log_2_ 45 = 5.492) are considered positive. Intermediate titres are considered as inconclusive (indicated within grey bar).

Neutralizing antibodies in selected serum samples were also measured using VNTs (Fig 7A–7C). These assays indicated that strong anti-FMDV neutralizing antibody responses were generated by the prime-boost strategy used in animals C4-C6 by PVD 28 and that the use of the same components, in the opposite order in animals C7-C9, was much less efficient (Fig 7B). Interestingly, the level of neutralizing antibodies induced by the FMDV challenge in the naïve animals (C1-C3, see Fig 7C) was not very different from that observed pre-challenge (PVD 28) in calves C4-C6 (rSFV-FMDV-primed and empty capsid-boosted). In calves C4-C6, there was little change in the level of neutralizing antibodies post-challenge (c.f. Fig 7B and 7C). In contrast, in calves C7-C9, there was a large increase in the level of neutralizing antibodies post-challenge (c.f. Fig 7B and 7C) consistent with the apparent FMDV infection.

## Discussion

Current vaccines to prevent FMD rely on the production of large quantities of infectious FMDV that are then chemically inactivated and injected, with an adjuvant, to induce protection against disease [7,8]. These vaccines have proved effective in controlling the disease in Europe but the disease is still endemic in many countries around the world, especially within Africa and parts of Asia. There are multiple, significant shortcomings with current FMD vaccines (see [9]) and hence there is a need to develop improved systems to allow better control of the disease. The focus here has been to express, within the cytoplasm, RNAs encoding the FMDV proteins required for the formation of empty capsid particles. It has been established that expression of the capsid precursor P1-2A along with a relatively low level of the 3C^pro^ (c.f. the viral polyprotein that yields equimolar amounts of the capsid precursor and 3C^pro^) is optimal to achieve efficient production of the FMDV empty capsid components [11,12,13,26] that can form the basis of improved vaccines.

The split-helper SFV vector system [23] generates virus particles that are able to initiate only a single round of virus infection. SFV replicates entirely within the cytoplasm of cells and produces a sub-genomic RNA that can be modified to express foreign antigens. In this study, rSFVs have been constructed to express the O1 Manisa FMDV capsid precursor (P1-2A) alone or with the FMDV 3C^pro^ (see Fig 1). The FMDV products had the expected physical properties and were able to bind both to FMDV antibodies and to the cellular receptor for FMDV (see Figs 3 and 4). Assembly of the processed viral proteins into rapidly sedimenting particles, consistent with the formation of empty capsid particles, was also demonstrated (Fig 4A).

It is known that serotype A FMDVs frequently generate empty capsid particles at a high level within FMDV-infected cells whereas the serotype O FMDVs do not normally show this property [10,39]. This may reflect differences in the stability of these particles and may contribute to the better success with the adenovirus vectors that express serotype A rather than serotype O capsid sequences [21]. The O1 Manisa strain, as used here, has been extensively employed for vaccine production and may be better in this respect than many other serotype O viruses [13].

Single cycle rSFV vectors that express the O1 Manisa capsid protein precursor alone and also with the 3C^pro^ have now been produced and characterized. Following a single inoculation of the rSFVs into cattle, the major target species for vaccination against FMD, an anti-FMDV response was induced but the level of antibodies produced was relatively low and proved insufficient to protect against challenge with a serotype O FMDV (into the tongue). However, the prior vaccination, with the rSFV-FMDV-P1-2A-mIRES-3C appeared to reduce the duration and level of viremia that occurred following the FMDV challenge. More significantly, it was also noted that a much higher level of anti-FMDV antibodies was generated, post-challenge, in the vaccinated animals than in the naïve controls (Table 1). This suggested that the rSFVs had primed the host immune response against the FMDV infection. This effect was observed in animals inoculated with the rSFV-FMDV-P1-2A and also, separately, with the rSFV-FMDV-P1-2A-mIRES-3C but the reduction in FMDV RNA in the serum was clearest with the rSFV-FMDV-P1-2A-mIRES-3C.

In a second experiment, which focused on the use of the rSFV-FMDV-P1-2A-mIRES-3C, it was found that a second vaccination with the same recombinant virus did not significantly improve the host immune response against FMDV (see S2 Fig and Table 2). It may be that the host response to the SFV particles induced by the primary vaccination blocked the ability of the rSFV to boost the anti-FMDV response. Indeed, the rSFV-FMDV-P1-2A-mIRES-3C was not detected (by RT-qPCR) in the serum of any of the animals following the 2^nd^ vaccination, while it was detected in nearly all animals following the primary inoculation (see Fig 5C and S2 Fig). These twice-vaccinated animals were not protected against FMDV infection, neither from direct inoculation (S2 Fig) nor by a more natural route of infection from another infected animal. However, once again a strong priming response was apparent from the anti-FMDV antibody titres (Table 2). These results suggested the potential utility of performing a two-stage vaccination process using the rSFV-FMDV in conjunction with purified FMDV empty capsid particles produced by another viral vector (as in [10,11]). The encapsidated rSFV-FMDV vector is technically fairly complex to produce and should be considered as a “proof of principle” for developing a cytoplasmic RNA vector based system for the expression of FMDV empty capsid particles within cells.

We have now shown that administration of the FMDV empty capsid particles after the initial vaccination with the rSFV-FMDV particles gave rise to a high level of anti-FMDV antibodies, prior to challenge. This immune response completely suppressed disease and the circulation of the virus in serum in all 3 calves (C4-C6) (see Fig 6H) and no lesions were observed away from the site of inoculation. It is noteworthy that the order of the inoculations is very important; the administration of the FMDV empty capsids followed by the rSFV-FMDV gave a much less effective immune response, pre-challenge, than the reverse order (Fig 6F and Table 3). The levels of circulating anti-FMDV antibodies were lower pre-challenge in all 3 calves (C7-C9) and significant viremia was detected post-challenge (although much lower than in unvaccinated animals) (Fig 6I). Furthermore, a much greater enhancement of anti-FMDV antibody levels was seen post-challenge (up to a titre of 1:5120) in group 3 compared to group 2 animals (see Table 3), presumably reflecting this higher level of FMDV replication. Similarly, the anti-FMDV titres measured by VNT greatly increased post-challenge in calves C7-C9 and also in the control group (calves C1-C3) but hardly changed in calves C4-C6 (Fig 7B and 7C).

It has been reported in an earlier study, that in cattle given two vaccinations with wt A22 empty capsid particles alone and then challenged with FMDV, all 4 animals showed viremia on at least one day [11]. Furthermore, 2 of 4 animals given two vaccinations with stabilized A22 empty capsids also showed viremia on 1 day post challenge. In the current study, in total some 30 cattle have been challenged with FMDV; viremia plus disease was observed in each of them except for the group that had received the prime-boost vaccination strategy employing the rSFV-FMDV-P1-2A-mIRES-3C followed by the empty capsid particles. The latter animals showed no disease; they had a complete absence of viremia and no spread of the virus from the site of inoculation was apparent.

It is noteworthy that current FMDV vaccines, although conferring protection against disease, do not provide sterile immunity [41]; indeed vaccinated and sub-clinically infected cattle are able to transmit the virus to other susceptible animals [42]. Future studies should address whether cattle, which have been vaccinated using the prime-boost strategy described here, are still able transmit the disease following virus challenge. Furthermore, it will also be important to determine the duration of the protective immune response generated by such vaccination regimes. An assessment of the role of the rSFV-FMDV in inducing cell-mediated immune responses will be a component of such studies. As indicated above, current control strategies can require vaccination 2–3 times per year since the duration of protection is rather limited. It is envisaged that any further boost of the immune response that may be required, following the prime-boost vaccination described here, could employ just the empty capsid particles and not require further priming with the rSFV-FMDV but this remains to be determined.

Both components of the prime-boost strategy described here can be produced outside of high-containment facilities and the system should be applicable to each of the FMDV serotypes. In addition, there is no requirement to adapt a particular FMDV strain for growth in cell culture to allow vaccine production.

## Supporting Information

S1 Fig Detection of expressed FMDV capsid proteins in bovine and porcine cells. Primary bovine thyroid cells (pBTY) (panels A and C) or porcine IBRS-2 cells (panels B and D) were infected with the indicated rSFVs. Cell lysates were prepared and analysed using SDS–PAGE and immunoblotting. The membranes were probed with antibodies specific for FMDV capsid proteins (panels A and B, top) and β-actin (panels A and B, bottom). Detection of β-actin was used as a control for equal protein loading. The results shown are representative of three independent experiments. Molecular mass markers (kDa) are indicated on the left. Cytoplasmic extracts were also analysed using an FMDV antigen ELISA (as in Fig 3). Cell lysates, (as used for panels A and B) were diluted (10-fold initially and then 2-fold dilutions) and analysed using an FMDV serotype O-specific antigen ELISA. The results shown are representative of two independent experiments. AU, absorbance units.
(TIF)

S2 Fig Effect of primary and secondary vaccination of cattle with rSFV-FMDV-P1-2A-mIRES-3C. In experiment 2, calves in group 1 (C1 and C2, see panels A, C and E) were unvaccinated. The animals in group 3 (C5-C7, see panels B, D, and F) were vaccinated with rSFV-FMDV-P1-2A-mIRES-3C on PVD 0 and again on PVD 14. The calves in groups 1 and 3 were challenged with FMDV by needle inoculation on PVD 28. Rectal temperatures were recorded on a daily basis. (see panels A, B) Serum was collected from each animal on the indicated days and assayed for anti-FMDV antibodies by blocking ELISA. The diagnostic cut-off level (50%) in the assay is indicated (see panels C, D). FMDV RNA in the sera from the indicated calves was measured by RT-qPCR and presented as RNA copies/ml as in Fig 5. A level of 10^7^ copies/ml is indicated by a horizontal line (see panels E, F).
(TIF)

## References

[1] Knight-Jones TJ, Rushton J (2013) The economic impacts of foot and mouth disease—what are they, how big are they and where do they occur? Prev Vet Med 112: 161–173. 10.1016/j.prevetmed.2013.07.013 23958457 PMC3989032

[2] Defra (2004) Animal Health and Welfare: FMD Data Archive. http://footandmouth.fera.defra.gov.uk/.

[3] Acharya R, Fry E, Stuart D, Fox G, Rowlands D, Brown F (1989) The three-dimensional structure of foot-and-mouth disease virus at 2.9 A resolution. Nature 337: 709–716. 2537470 10.1038/337709a0

[4] Jackson T, Sheppard D, Denyer M, Blakemore W, King AM (2000) The epithelial integrin alphavbeta6 is a receptor for foot-and-mouth disease virus. J Virol 74: 4949–4956. 10799568 10.1128/jvi.74.11.4949-4956.2000 PMC110846

[5] Monaghan P, Gold S, Simpson J, Zhang Z, Weinreb PH, Violette SM et al (2005) The alpha(v)beta6 integrin receptor for Foot-and-mouth disease virus is expressed constitutively on the epithelial cells targeted in cattle. J Gen Virol 86: 2769–2780. 16186231 10.1099/vir.0.81172-0

[6] Belsham GJ (2005) Translation and replication of FMDV RNA. Curr Top Microbiol Immunol 288: 43–70. 15648174 10.1007/3-540-27109-0_3

[7] Doel TR (2003) FMD vaccines. Virus Res 91: 81–99. 12527439 10.1016/s0168-1702(02)00261-7

[8] Rodriguez LL, Grubman MJ (2009) Foot and mouth disease virus vaccines. Vaccine 27 Suppl 4: D90–94. 10.1016/j.vaccine.2009.08.039 19837296

[9] Belsham GJ, Bøtner A (2015) Use of recombinant capsid proteins in the development of a vaccine against the foot-and-mouth disease virus (FMDV). Virus Adaptation and Treatment 7: 1–13.

[10] Abrams CC, King AM, Belsham GJ (1995) Assembly of foot-and-mouth disease virus empty capsids synthesized by a vaccinia virus expression system. J Gen Virol 76: 3089–3098. 8847514 10.1099/0022-1317-76-12-3089

[11] Porta C, Kotecha A, Burman A, Jackson T, Ren J, Loureiro S et al (2013) Rational engineering of recombinant picornavirus capsids to produce safe, protective vaccine antigen. PLoS Pathog 9: e1003255 10.1371/journal.ppat.1003255 23544011 PMC3609824

[12] Porta C, Xu X, Loureiro S, Paramasivam S, Ren J, Al-Khalil et al (2013) Efficient production of foot-and-mouth disease virus empty capsids in insect cells following down regulation of 3C protease activity. J Virol Methods 187: 406–412. 10.1016/j.jviromet.2012.11.011 23174161 PMC3558679

[13] Gullberg M, Muszynski B, Organtini LJ, Ashley RE, Hafenstein SL, Belsham GJ et al (2013) Assembly and characterization of foot-and-mouth disease virus empty capsid particles expressed within mammalian cells. J Gen Virol 94: 1769–1779. 10.1099/vir.0.054122-0 23740480

[14] Chow M, Newman JF, Filman D, Hogle JM, Rowlands DJ, Brown F (1987) Myristylation of picornavirus capsid protein VP4 and its structural significance. Nature 327: 482–486. 3035380 10.1038/327482a0

[15] Ansardi DC, Porter DC, Morrow CD (1992) Myristylation of poliovirus capsid precursor P1 is required for assembly of subviral particles. J Virol 66: 4556–4563. 1318418 10.1128/jvi.66.7.4556-4563.1992 PMC241268

[16] Ellard FM, Drew J, Blakemore WE, Stuart DI, King AM (1999) Evidence for the role of His-142 of protein 1C in the acid-induced disassembly of foot-and-mouth disease virus capsids. J Gen Virol 80: 1911–1918. 10466786 10.1099/0022-1317-80-8-1911

[17] Mateo R, Luna E, Rincon V, Mateu MG (2008) Engineering viable foot-and-mouth disease viruses with increased thermostability as a step in the development of improved vaccines. J Virol 82: 12232–12240. 10.1128/JVI.01553-08 18829763 PMC2593342

[18] Rincon V, Rodriguez-Huete A, Lopez-Arguello S, Ibarra-Molero B, Sanchez-Ruiz JM, Harmsen MM et al (2014) Identification of the structural basis of thermal lability of a virus provides a rationale for improved vaccines. Structure 22: 1560–1570. 10.1016/j.str.2014.08.019 25308865

[19] Caridi F, Vazquez-Calvo A, Sobrino F, Martin-Acebes MA (2015) The pH stability of foot-and-mouth disease virus particles is modulated by residues located at the pentameric interface and in the N terminus of VP1. J Virol 89: 5633–5642. 10.1128/JVI.03358-14 25762735 PMC4442502

[20] Moraes MP, Mayr GA, Mason PW, Grubman MJ (2002) Early protection against homologous challenge after a single dose of replication-defective human adenovirus type 5 expressing capsid proteins of foot-and-mouth disease virus (FMDV) strain A24. Vaccine 20: 1631–1639. 11858872 10.1016/s0264-410x(01)00483-2

[21] Moraes MP, Segundo FD, Dias CC, Pena L, Grubman MJ (2011) Increased efficacy of an adenovirus-vectored foot-and-mouth disease capsid subunit vaccine expressing nonstructural protein 2B is associated with a specific T cell response. Vaccine 29: 9431–9440. 10.1016/j.vaccine.2011.10.037 22027486

[22] Karlsson GB, Liljestrom P (2003) Live viral vectors: Semliki Forest virus. Methods Mol Med 87: 69–82. 12958450 10.1385/1-59259-399-2:69

[23] Smerdou C, Liljestrom P (1999) Two-helper RNA system for production of recombinant Semliki forest virus particles. J Virol 73: 1092–1098. 9882310 10.1128/jvi.73.2.1092-1098.1999 PMC103929

[24] Sambrook J, Fritsch EF, Maniatis T (1989) Molecular cloning: a laboratory manual Cold Spring Harbor NY Cold Spring Harbor Laboratory.

[25] Liljestrom P, Garoff H (1991) A new generation of animal cell expression vectors based on the Semliki Forest virus replicon. Biotechnology (N Y) 9: 1356–1361. 1370252 10.1038/nbt1291-1356

[26] Polacek C, Gullberg M, Li J, Belsham GJ (2013) Low levels of foot-and-mouth disease virus 3C protease expression are required to achieve optimal capsid protein expression and processing in mammalian cells. J Gen Virol 94: 1249–1258. 10.1099/vir.0.050492-0 23364188

[27] Bøtner A, Kakker NK, Barbezange C, Berryman S, Jackson T, Belsham GJ (2011) Capsid proteins from field strains of foot-and-mouth disease virus confer a pathogenic phenotype in cattle on an attenuated, cell-culture-adapted virus, O1 Kaufbeuren virus. J Gen Virol 92: 1141–1151. 10.1099/vir.0.029710-0 21270284

[28] Nayak A, Goodfellow IG, Woolaway KE, Birtley J, Curry S, Belsham GJ (2006) Role of RNA structure and RNA binding activity of foot-and-mouth disease virus 3C protein in VPg uridylylation and virus replication. J Virol 80: 9865–9875. 16973591 10.1128/JVI.00561-06 PMC1617274

[29] Gullberg M, Polacek C, Bøtner A, Belsham GJ (2013) Processing of the VP1/2A junction is not necessary for production of foot-and-mouth disease virus empty capsids and infectious viruses: characterization of "self-tagged" particles. J Virol 87: 11591–11603. 10.1128/JVI.01863-13 23966400 PMC3807367

[30] Belsham GJ, McInerney GM, Ross-Smith N (2000) Foot-and-mouth disease virus 3C protease induces cleavage of translation initiation factors eIF4A and eIF4G within infected cells. J Virol 74: 272–280. 10590115 10.1128/jvi.74.1.272-280.2000 PMC111537

[31] OIE (2012) Foot-and-mouth disease. Manual of diagnostic tests and vaccines for terrestrial animals (mammals, birds and bees). http://wwwoieint/fileadmin/Home/eng/Health_standards/tahm/20105_FMDpdf1. 16642778

[32] Roeder PL, Le Blanc Smith PM (1987) Detection and typing of foot-and-mouth disease virus by enzyme-linked immunosorbent assay: a sensitive, rapid and reliable technique for primary diagnosis. Res Vet Sci 43: 225–232. 2825310

[33] Stenfeldt C, Heegaard PM, Stockmarr A, Tjørnehøj K, Belsham GJ (2011) Analysis of the acute phase responses of serum amyloid a, haptoglobin and type 1 interferon in cattle experimentally infected with foot-and-mouth disease virus serotype O. Vet Res 42: 66 10.1186/1297-9716-42-66 21592356 PMC3123197

[34] Fuerst TR, Niles EG, Studier FW, Moss B (1986) Eukaryotic transient-expression system based on recombinant vaccinia virus that synthesizes bacteriophage T7 RNA polymerase. Proc Natl Acad Sci U S A 83: 8122–8126. 3095828 10.1073/pnas.83.21.8122 PMC386879

[35] Kotecha A, Seago J, Scott K, Burman A, Loureiro S, Ren J et al (2015) Structure-based energetics of protein interfaces guides foot-and-mouth disease virus vaccine design. Nat Struct Mol Biol 22: 788–794. 10.1038/nsmb.3096 26389739 PMC5985953

[36] Reid SM, Ferris NP, Hutchings GH, Zhang Z, Belsham GJ, Alexandersen S (2002) Detection of all seven serotypes of foot-and-mouth disease virus by real-time, fluorogenic reverse transcription polymerase chain reaction assay. J Virol Methods 105: 67–80. 12176143 10.1016/s0166-0934(02)00081-2

[37] Balinda SN, Tjørnehøj K, Muwanika VB, Sangula AK, Mwiine FN, Ayebazibwe C et al (2009) Prevalence estimates of antibodies towards foot-and-mouth disease virus in small ruminants in Uganda. Transbound Emerg Dis 56: 362–371. 10.1111/j.1865-1682.2009.01094.x 19909475

[38] Sweeney TR, Roque-Rosell N, Birtley JR, Leatherbarrow RJ, Curry S (2007) Structural and mutagenic analysis of foot-and-mouth disease virus 3C protease reveals the role of the beta-ribbon in proteolysis. J Virol 81: 115–124. 17065215 10.1128/JVI.01587-06 PMC1797255

[39] Rweyemamu MM, Terry G, Pay TW (1979) Stability and immunogenicity of empty particles of foot-and-mouth disease virus. Arch Virol 59: 69–79. 218538 10.1007/BF01317896

[40] Mayr GA, O'Donnell V, Chinsangaram J, Mason PW, Grubman MJ (2001) Immune responses and protection against foot-and-mouth disease virus (FMDV) challenge in swine vaccinated with adenovirus-FMDV constructs. Vaccine 19: 2152–2162. 11228388 10.1016/s0264-410x(00)00384-4

[41] Barnett PV, Carabin H (2002) A review of emergency foot-and-mouth disease (FMD) vaccines. Vaccine 20: 1505–1514. 11858856 10.1016/s0264-410x(01)00503-5

[42] Donaldson AI, Kitching RP (1989) Transmission of foot-and-mouth disease by vaccinated cattle following natural challenge. Res Vet Sci 46: 9–14. 2537993

