# Correction: A Prime-Boost Vaccination Strategy in Cattle to Prevent Foot-and-Mouth Disease Using a “Single-Cycle” Alphavirus Vector and Empty Capsid Particles

**DOI:** 10.1371/journal.pone.0169025

**Published:** 2016-12-20

**Authors:** The PLOS ONE Staff

There is an error in Table 3. The values in columns Post-prime, Post-boost and Post-challenge were incorrectly omitted. The authors have provided a corrected version here. The publisher apologizes for the error.

**Table 3:** Reciprocal titres of anti-FMDV antibodies (serotype O) in sera from calves in experiment 3.

			Post-prime	Post-boost	Post-challenge
Group	Vaccination	Animal	PVD 14	PVD 28	PVD 36
1	No vaccination (control)	C1	-	-	40
		C2	-	-	40
		C3	-	-	160
2	rSFV-FMDV-P1-2A-mIRES-3C (PVD 0) + empty capsid particles (PVD 14)	C4	-	320	1280
		C5	-	40	320
		C6	20	320	1280
3	empty capsid particles (PVD 0) + rSFV-FMDV-P1-2A-mIRES-3C (PVD 14)	C7	20	20	5120
		C8	-	80	5120
		C9	-	10	5120

Calves were either unvaccinated (group 1) or vaccinated with rSFV-FMDV-P1-2A-mIRES-3C (on PVD 0) followed by empty capsid particles (on PVD 14) (group 2) or vaccinated with empty capsids on PVD 0 and then with rSFV-FMDV-P1-2A-mIRES-3C on PVD 14 (group 3). All cattle were challenged with FMDV by needle inoculation on PVD 28. Sera collected on PVD 14, 28 (pre-challenge) and 36 were titred in the blocking ELISA using 2-fold dilutions starting at 1:5.—indicates negative.
