# Correction: A Prime-Boost Vaccination Strategy in Cattle to Prevent Foot-and-Mouth Disease Using a "Single-Cycle" Alphavirus Vector and Empty Capsid Particles

**DOI:** 10.1371/journal.pone.0173327

**Published:** 2017-03-01

**Authors:** Maria Gullberg, Louise Lohse, Anette Bøtner, Gerald M. McInerney, Alison Burman, Terry Jackson, Charlotta Polacek, Graham J. Belsham

There is an error in Table 1. The values in the last two rows for Animals C7 and C8 in Group 3 were incorrectly omitted. The authors have provided a corrected version here.

**Table 1:** Reciprocal titres of anti-FMDV antibodies (serotype O) in sera from unvaccinated and rSFV-FMDV vaccinated calves in experiment 1.

			Pre-challenge		Post-challenge	
Group	Vaccination and challenge	Animal	PVD 14	PVD 21	PVD 27	PVD 30
1	No vaccination (control)	C1	-	-	-	20
	Needle challenge	C2	-	-	-	20
2	rSFV-FMDV-P1-2A	C3	-	-	320	320
	Needle challenge	C4	-	-	320	320
		C5	-	5	640	320
3	rSFV-FMDV-P1-2A-mIRES-3C	C6	5	10	320	320
	Needle challenge	C7	-	-	320	320
		C8	-	-	320	320

Calves C3 to C8 were vaccinated with the indicated rSFV-FMDV on PVD 0 and then challenged with FMDV by needle inoculation on PVD 21. To determine the titre, sera collected from calves on PVD 14, 21, 27 and 30 were assayed in the ELISA using 2-fold dilutions starting at 1:5.—indicates negative.
