# Supplementary figures and images for: A Prime-Boost Vaccination Strategy in Cattle to Prevent Foot-and-Mouth Disease Using a “Single-Cycle” Alphavirus Vector and Empty Capsid Particles
Source: PLoS One. 2016 Jun 13;11(6):e0157435. doi: 10.1371/journal.pone.0157435 (PMC4905628; doi:10.1371/journal.pone.0157435)

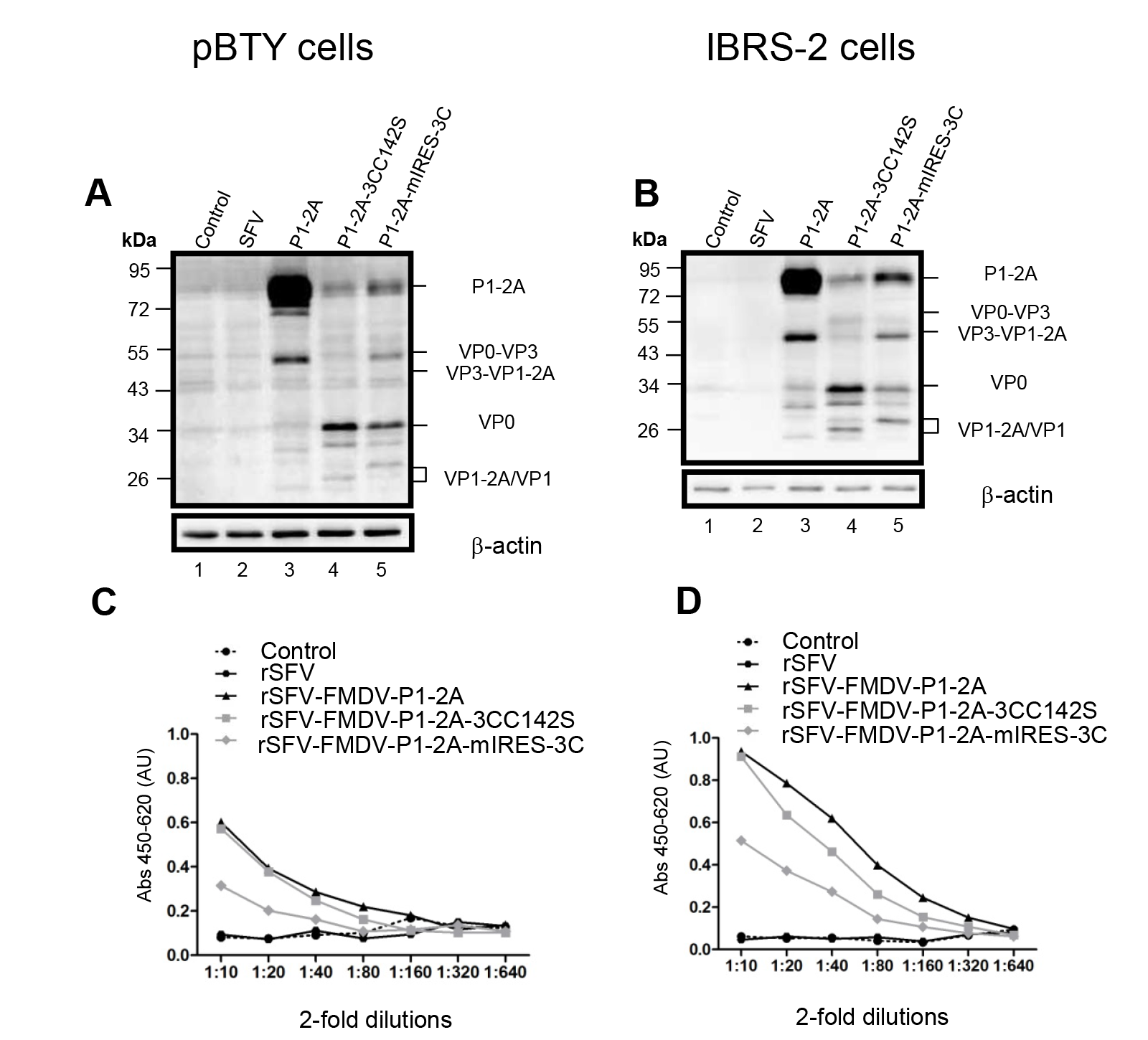

Supplement: S1 Fig — Primary bovine thyroid cells (pBTY) (panels A and C) or porcine IBRS-2 cells (panels B and D) were infected with the indicated rSFVs. Cell lysates were prepared and analysed using SDS–PAGE and immunoblotting. The membranes were probed with antibodies specific for FMDV capsid proteins (panels A and B, top) and β-actin (panels A and B, bottom). Detection of β-actin was used as a control for equal protein loading. The results shown are representative of three independent experiments. Molecular mass markers (kDa) are indicated on the left. Cytoplasmic extracts were also analysed using an FMDV antigen ELISA (as in Fig 3). Cell lysates, (as used for panels A and B) were diluted (10-fold initially and then 2-fold dilutions) and analysed using an FMDV serotype O-specific antigen ELISA. The results shown are representative of two independent experiments. AU, absorbance units. (TIF) [file pone.0157435.s001.tif]

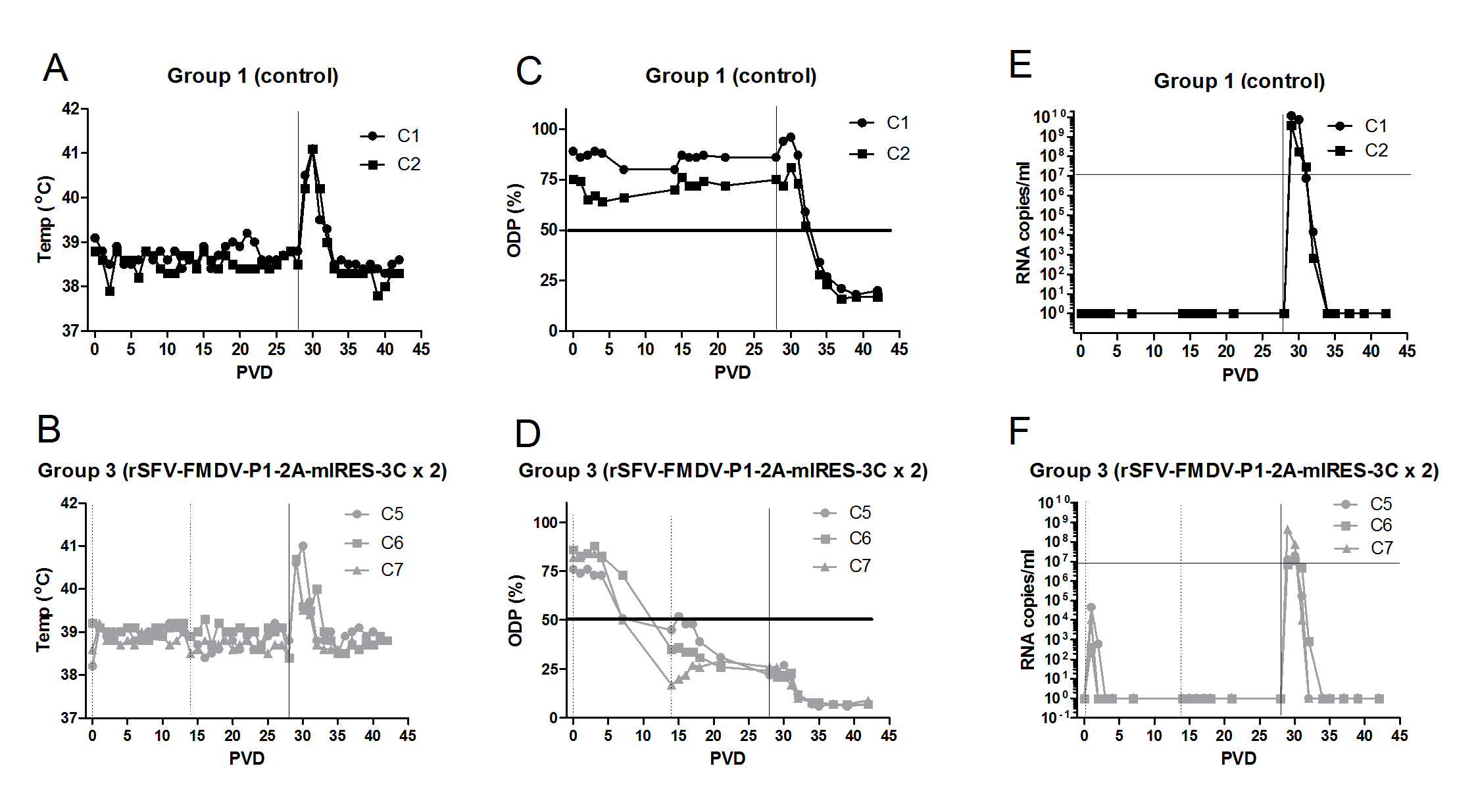

Supplement: S2 Fig — In experiment 2, calves in group 1 (C1 and C2, see panels A, C and E) were unvaccinated. The animals in group 3 (C5-C7, see panels B, D, and F) were vaccinated with rSFV-FMDV-P1-2A-mIRES-3C on PVD 0 and again on PVD 14. The calves in groups 1 and 3 were challenged with FMDV by needle inoculation on PVD 28. Rectal temperatures were recorded on a daily basis. (see panels A, B) Serum was collected from each animal on the indicated days and assayed for anti-FMDV antibodies by blocking ELISA. The diagnostic cut-off level (50%) in the assay is indicated (see panels C, D). FMDV RNA in the sera from the indicated calves was measured by RT-qPCR and presented as RNA copies/ml as in Fig 5. A level of 107 copies/ml is indicated by a horizontal line (see panels E, F). (TIF) [file pone.0157435.s002.tif]
